# Alternative splicing of *OsLG3b* controls grain length and yield in *japonica* rice

**DOI:** 10.1111/pbi.12903

**Published:** 2018-03-24

**Authors:** Jianping Yu, Jinli Miao, Zhanying Zhang, Haiyan Xiong, Xiaoyang Zhu, Xingming Sun, Yinghua Pan, Yuntao Liang, Qiang Zhang, Rashid Muhammad Abdul Rehman, Jinjie Li, Hongliang Zhang, Zichao Li

**Affiliations:** ^1^ Key Laboratory of Crop Heterosis and Utilization Ministry of Education/Beijing Key Laboratory of Crop Genetic Improvement China Agricultural University Beijing China; ^2^ China/Guangxi Key Laboratory of Rice Genetics and Breeding Rice Research Institute Guangxi Academy of Agricultural Sciences Nanning, Guangxi China

**Keywords:** artificial selection, rice domestication, linkage mapping, *Oryza sativa*, Alternative splicing, *OsLG3b*, grain length

## Abstract

Grain size, one of the important components determining grain yield in rice, is controlled by the multiple quantitative trait loci (QTLs). Intensive artificial selection for grain size during domestication is evidenced in modern cultivars compared to their wild relatives. Here, we report the molecular cloning and characterization of *OsLG3b*, a QTL for grain length in tropical *japonica* rice that encodes MADS‐box transcription factor 1 (OsMADS1). Six SNPs in the *OsLG3b* region led to alternative splicing, which were associated with grain length in an association analysis of candidate region. Quantitative PCR analysis indicated that *OsLG3b* expression was higher during the panicle and seed development stages. Analysis of haplotypes and introgression regions revealed that the long‐grain allele of *OsLG3b* might have arisen after domestication of tropical *japonica* and spread to subspecies *indica* or temperate *japonica* by natural crossing and artificial selection. *OsLG3b* is therefore a target of human selection for adaptation to tropical regions during domestication and/or improvement of rice. Phylogenetic analysis and pedigree records showed that *OsLG3b* had been employed by breeders, but the gene still has much breeding potential for increasing grain length in *indica*. These findings will not only aid efforts to elucidate the molecular basis of grain development and domestication, but also facilitate the genetic improvement of rice yield.

## Introduction

Rice is one of staple food crops for over half of the world's population. Grain size, grain number and panicle number are the three component traits that determine rice yield. Among them, grain size is considered the main breeding target for its effect on both yield and quality, which is limited by grain length, width and thickness of the grain. Therefore, it is important to study grain size for improving yield and quality as well as understanding the domestication process that has occurred in rice (Shomura *et al*., 2008). Although a number of quantitative trait loci (QTLs) conferring grain length (Huang *et al*., 2012a) have been isolated, only a few genes have been well studied, such as *GS3*, *GW8, GL3.1*, *OsLG3, TGW6*, *qSW5* and *GW2* (Ishimaru *et al*., 2013; Mao *et al*., 2010; Shomura *et al*., 2008; Song *et al*., 2007; Wang *et al*., 2012; Yu *et al*., 2017; Zhang *et al*., 2012b). Therefore, the genetic dissection and molecular characterization of more genes conferring grain length need to be conducted (Mao *et al*., 2010).

During the evolution of cultivated rice, grain size is usually affected by artificial selection. Thus, isolation of novel genes conferring grain size will provide more evidence for the origin and evolution of cultivated rice. Lately several QTLs controlling grain size that were selected during domestication have been cloned. *Grain size 3* (*GS3*) is the first cloned QTL that negatively regulates grain length (Fan *et al*., 2006; Takano‐Kai *et al*., 2009). The C165A mutation in the exon 2 was a functional mutation widely spread in rice germplasm (Fan *et al*., 2009; Wang *et al*., 2011). This mutation arose from an ancient *japonica* and then flowed into the *indica* by introgression (Takano‐Kai *et al*., 2009). The QTL for *grain width 5 (GW5/qSW5/GSE5)* was a key gene involved in *japonica* rice domestication and natural variation in its promoter region contributed to diversity of grain size. Both the DEL1 (the 950‐bp deletion) and the DEL2 (the 1212‐bp deletion) were thought to be selected during the propagation of cultivation areas for *indica and japonica*, respectively (Duan *et al*., 2017; Liu *et al*., 2017; Shomura *et al*., 2008; Weng *et al*., 2008). Another gene, *GRAIN NUMBER, GRAIN LENGTH AND AWN DEVELOPMENT 1 (GAD1),* encodes a small secretary signal peptide that belongs to the EPIDERMAL PATTERNING FACTOR‐LIKE family (Jin *et al*., 2016). This locus was strongly selected in *O. sativa* during domestication. All of these genes might help to understand the origin and domestication of Asian cultivated rice at the molecular level.

Slender grains with a large ratio of length‐to‐width are preferred by the majority of consumers (Fan *et al*., 2006), especially in South‐East Asia. The traditional tropical *japonica*, generally grown in tropical and subtropical regions, usually has a more slender and larger grain than those of temperate *japonica* (Figure S1 and S12 and Table S1). The genetic basis of these differences requires further investigation. In this research, we identified an important QTL, *OsLG3b,* controlling grain length and weight in tropical *japonica* rice by map‐based cloning. We used an association study to demonstrate that natural variation in *OsLG3b* was significantly associated with grain length, and this was confirmed by genetic transformation. We also identified the origin of the variations leading to increased grain length in tropical *japonica* and found evidence of selection across the *OsLG3b* region. The elite allele of *OsLG3b* could be used to breed new elite varieties.

## Results

### QTLs for grain size detected by linkage mapping

**Figure 1:**
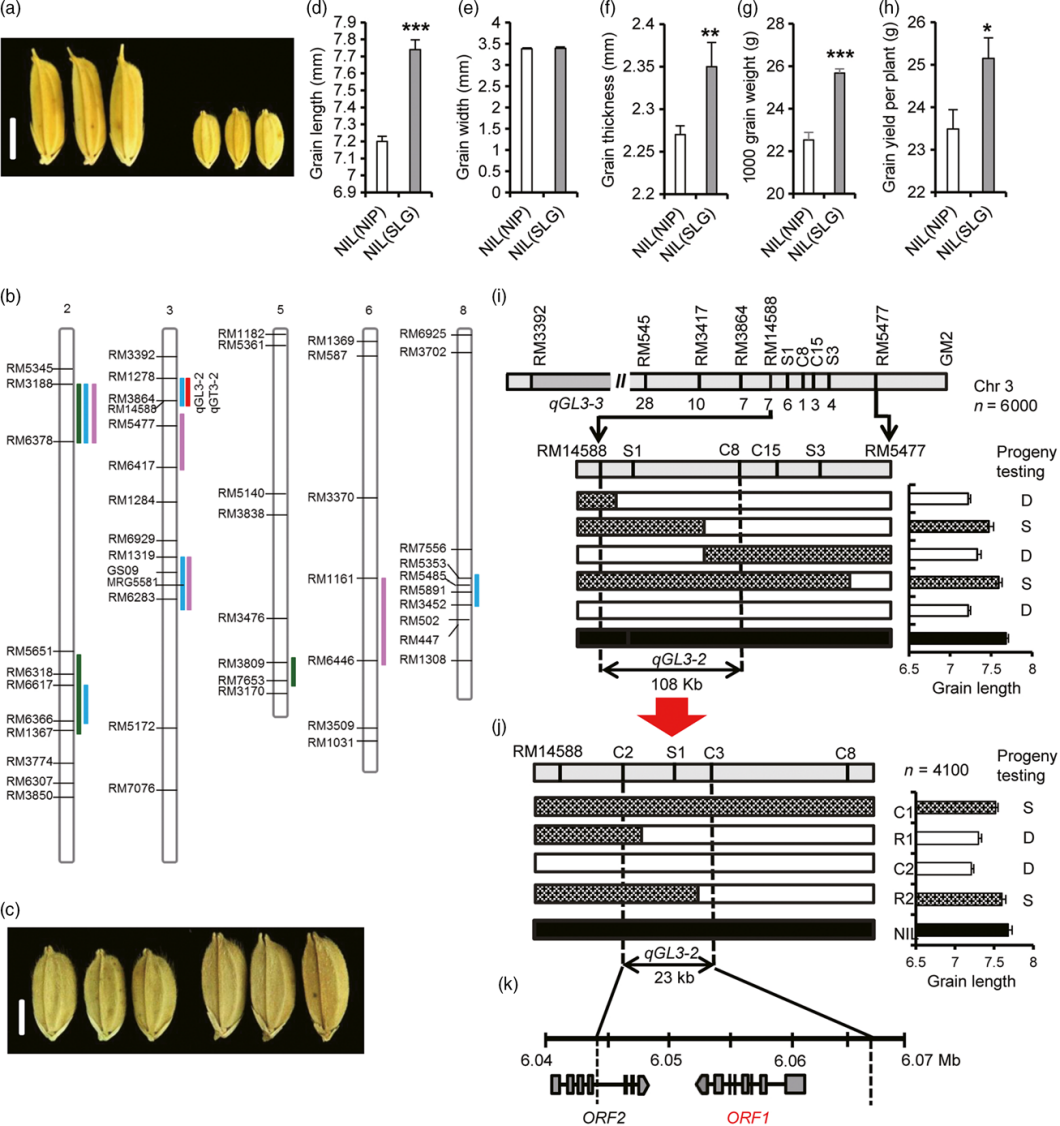
Map‐based cloning of *OsLG3b*. (a) Grain morphology of SLG and NIP. Scale bar, 5 mm. (b) QTL locations for grain length (GL, blue bars), grain width (GW, green bars), grain thickness (GT, red bars) and 1000 grain weight (TGW, pink bars). (c) NIL(NIP) and NIL(SLG) grains. Scale bar, 2.5 mm. (d) Grain length, (e) grain width, (f) grain thickness, (g) 1000 grain weight, (h) grain yield per plant. All phenotypic data in d–h were measured on paddy‐grown plants. Data represent mean ± S.E.M. (*n* = 10). Student's *t*‐tests were used to determine *P* values. *P < 0.05, **P < 0.01. (i, j) High‐resolution linkage analysis of phenotypes and marker genotypes. White bars represent chromosomal segments for Nipponbare homozygote, black for SLG homozygote, and grille for heterozygotes. Genotypes at the *qGL3‐2* locus were confirmed by progeny tests. S, segregation; D, desegregation. (k) Predicted ORFs based on the Nipponbare genome sequence. *ORF1*, *Os03g0215400*; *ORF2*, *Os03g0215200*.

SLG‐1 (SLG), one of the varieties with the largest grain (1000 grain weight: 58.8 ± 1.07 g), is an improved temperate *japonica* developed from crosses involving *tropical japonica*. In contrast, Nipponbare (NIP), a typical temperate *japonica*, has small grain (1000 grain weight: 23.3 ± 0.2 g; Figure 1a). QTL analyses were carried out using BC_4_F_2_ and BC_4_F_3_ populations derived from a cross between SLG and NIP to analyse the molecular basis of large grain in SLG. Five QTLs for grain length, three for grain width, one for grain thickness and four for grain weight were detected (Table S2). Among them, *qGW2‐1* (LOD score = 12.27), *qGL3‐1* (LOD score = 26.68) and *qTGW6‐1* (LOD score = 2.64) were mapped to the same regions as three reported genes, *GW2, GS3* and *TGW6*, respectively (Figure 1b; Table S2). Sequencing data for these three genes confirmed allelic differences between SLG and NIP (Table S3). Importantly, we identified a grain length QTL, *qGL3‐2*, on the long arm of chromosome 3 (Figure 1b), explaining 18.0% (LOD score = 8.0) of the phenotypic variation in grain length and 17.2% (LOD score = 7.3) of the variation in grain thickness (Table S2). These results indicated that SLG pyramids at least three known large‐grain alleles (*gw2, gs3* and *TGW6*) and *qGL3‐2*.

### Fine mapping of *qGL3‐2*

To clone the gene for *qGL3‐2*, a near‐isogenic line was developed in the NIP background for the *qGL3‐2* locus, NIL(SLG), through repeated backcrossing with NIP (Figure S2). Grains of NIL(SLG) were 7.5% longer, and 3.5% thicker than those of NIP, leading to a 7.1% increase in grain yield per plant (Figure 1c‐h and Figure S3e, f, h and i). No significant differences were detected in other agronomic traits, such as heading date, number of tillers per plant and grain number per panicle (Figure S3a‐d and g). The *qGL3‐2* locus was preliminarily mapped to a 108‐kb region between RM14588 and C8 using 6000 BC_5_F_2_ plants derived from NIL (SLG) (Figure 1i). Another 4100 BC_5_F_3_ plants derived from BC_5_F_2_ recombinants were used to further narrow down the region to a 23‐kb interval flanked by the markers C2 and C3 (Figure 1j) that includes ORF1 (*Os03g0215400*) and the last three exons of ORF2 (Figure 1k). There was no polymorphism between the two parents in the coding sequence of ORF2, indicating that ORF1 was the most likely candidate gene for *qGL3‐2*. It was designated as *Oryza sativa long grain 3b* (hereafter *OsLG3b*). *OsLG3b* encodes MADS‐box transcription factor 1 (OsMADS1) controlling differentiation of specific cell types in the lemma and palea (Prasad *et al*., 2005).

### Variation in *OsLG3b* is significantly associated with grain length

**Figure 2:**
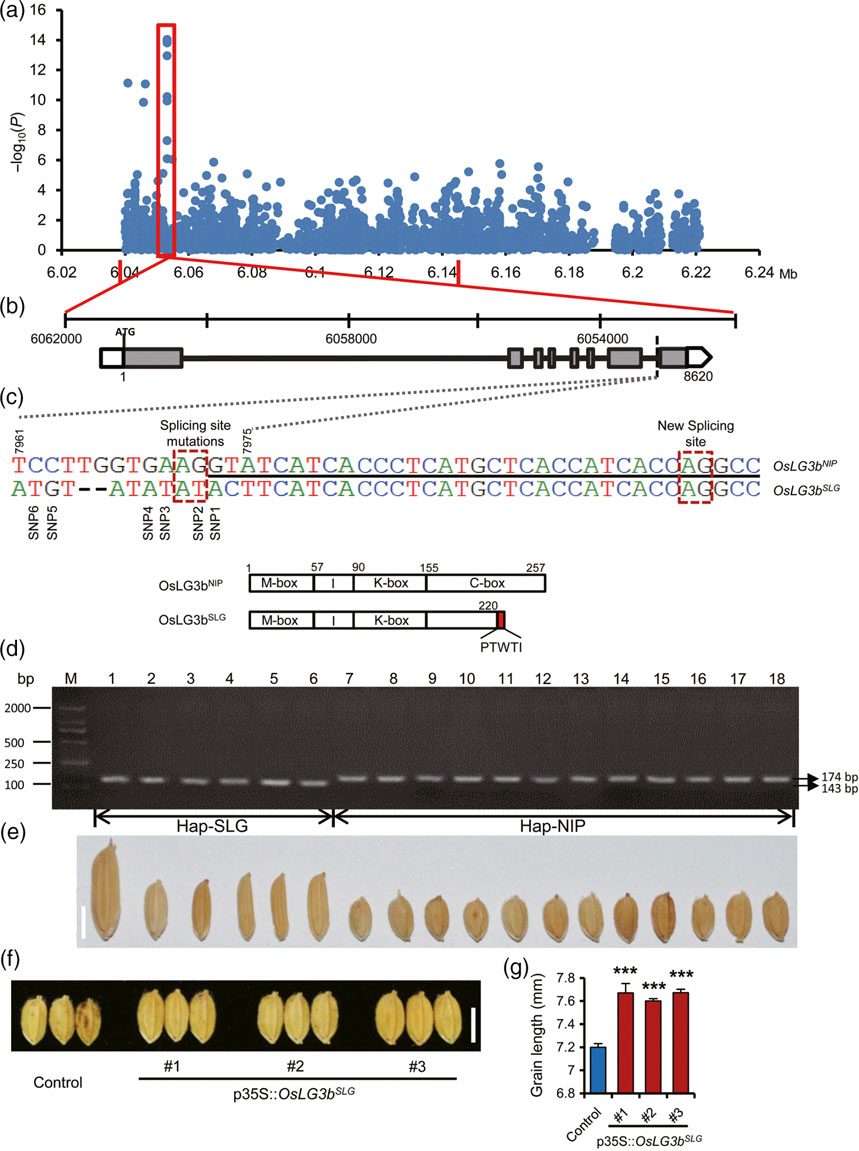
Identification of *OsLG3b* by association mapping. (a) Manhattan plot for candidate region association mapping of *qGL3‐2*. The two red vertical lines indicate the boundaries of *qGL3‐2*. (b) Gene structure of *Os03g0215400*. Empty boxes refer to 5′ and 3′‐UTRs, grey boxes are exons, and lines between boxes are introns. The variations (TCCTTGGTGAAGGTA → ATGT ‐ ‐ ATATATACT) in the *Os03g0215400* ^*SLG*^ are shown by dashed lines. (c) Structure of splicing site and mutation sites. Underlined nucleotides are the sequence of the first half of exons 8 in NIP; others are at the terminal of the intron 7. Snp1‐6, refer to six SNPs significantly associated with grain length (*P* < 1.0 × 10^−8^). In the schematic illustration of OsLG3b functional domains, SLG produced an alternatively spliced protein OsLG3b ^SLG^ , in which the terminal 37 residues were truncated and additional 5 residues were added to the predicted C domain. (d) Genotyping analyses of cDNA fragment encoded by the SLG and NIP alleles in 18 rice cultivars. We designed a pair of primers (Table S11) to amplify a small cDNA sequence that contains the splicing mutation region and the PCR fragment of SLG had a 31 nucleotides deletion. Therefore, varieties with the SLG allele form 143‐bp PCR products and varieties with the NIP allele form 174‐bp PCR products. (e) Grains of 18 rice cultivars. (f, g) Functional validation of *OsLG3b* by genetic transformation. (f) Grain shape in transgenic plants compared to control grains. Scale bar, 5 mm. (g) Comparison of grain length between transgenic plants and control. Data represent mean ± S.E.M. (*n* = 30). Student's *t*‐tests were used to generate *P* values.

In order to investigate natural variation in the mapping region of QTL *qGL3‐2*, independently of the fine‐mapping analysis described above, a candidate region association analysis was conducted on the mapped 108‐kb region of *qGL3‐2*. This approach utilized a mini‐core collection (MCC) (Zhang *et al*., 2011) panel (Figure S4 and Tables S5 and S6) of 266, as part of “The 3000 rice genomes project” (the rice 3k genome), which had been deeply sequenced (see URLs). High‐density SNPs (one SNP per 40 bp on average) were obtained (Yu *et al*., 2017). We measured the grain length of each variety grown in five circumstances (Table S7) and observed substantial and highly significant variation in grain size, with high heritability values varying from 82% to 97% and an average of 89% (Figure S5 and Table S8). To reduce the incidence of false positives, a general linear model (GLM) that controls for population structure (Q matrix) was used to identify significant genotypic and phenotypic associations. The association analysis detected six SNPs within *OsLG3b* that were significantly associated with grain length (*P* < 1.0 × 10^−8^) (Figure 2a, c and Table S9). One of these SNPs was found in the last exon, and five SNPs were in the last intron of the *Os03g0215400*.

To investigate functional variation, we sequenced *OsLG3b* and its upstream region in SLG (long grain) and NIP (short grain). Sequence comparison revealed no polymorphism in the promoter region, but there were 15 polymorphisms in the gene (both exons and introns), including four nucleotide substitutions in intron 1, three nucleotide substitutions at the start point of exon 8 and seven nucleotide substitutions and a 2‐bp deletion at the terminal of intron 7 (Figure 2b, c). These variations resulted in an alternative splicing event (AG/GT → AT/AC), and the splice site shifted to the 32nd nucleotide (AG/GC) of exon 8, introducing a premature stop codon and preventing transcription of a mature protein (Figure 2c and S8). The premature stop codon led to truncation of 32 amino acid residues, and the remaining portion of the protein consisted of a 225‐residue polypeptide (Figure S6). This result was validated by genotyping analyses of cDNA fragments encoded by the SLG and NIP alleles in 18 rice cultivars (Figure 2d, e). We also analysed the sequence of *OsLG3b* from IRAT109, a representative *tropical japonica* variety with long grain similar to SLG (Figure S7). Its sequence was identical to the SLG allele (Figures S6 and S8). These data indicated that the change in the coding region of *OsLG3b* was most likely responsible for the SLG grain phenotype.

Given that the promoter sequences of *OsLG3b* in SLG and NIP were the same, to determine whether the *OsLG3b* gene underlies the QTL, we introduced SLG allele *OsLG3b* cDNA into NIP under the control of a CaMV35S promoter. The grain length was restored to the NIL‐SLG level in transgenic plants (T_1_ generation) (Figure 2f, g), indicating that the change in function of *OsLG3b* resulted in increased grain length.

### Expression pattern of *OsLG3b* and its transcription activator activity

**Figure 3:**
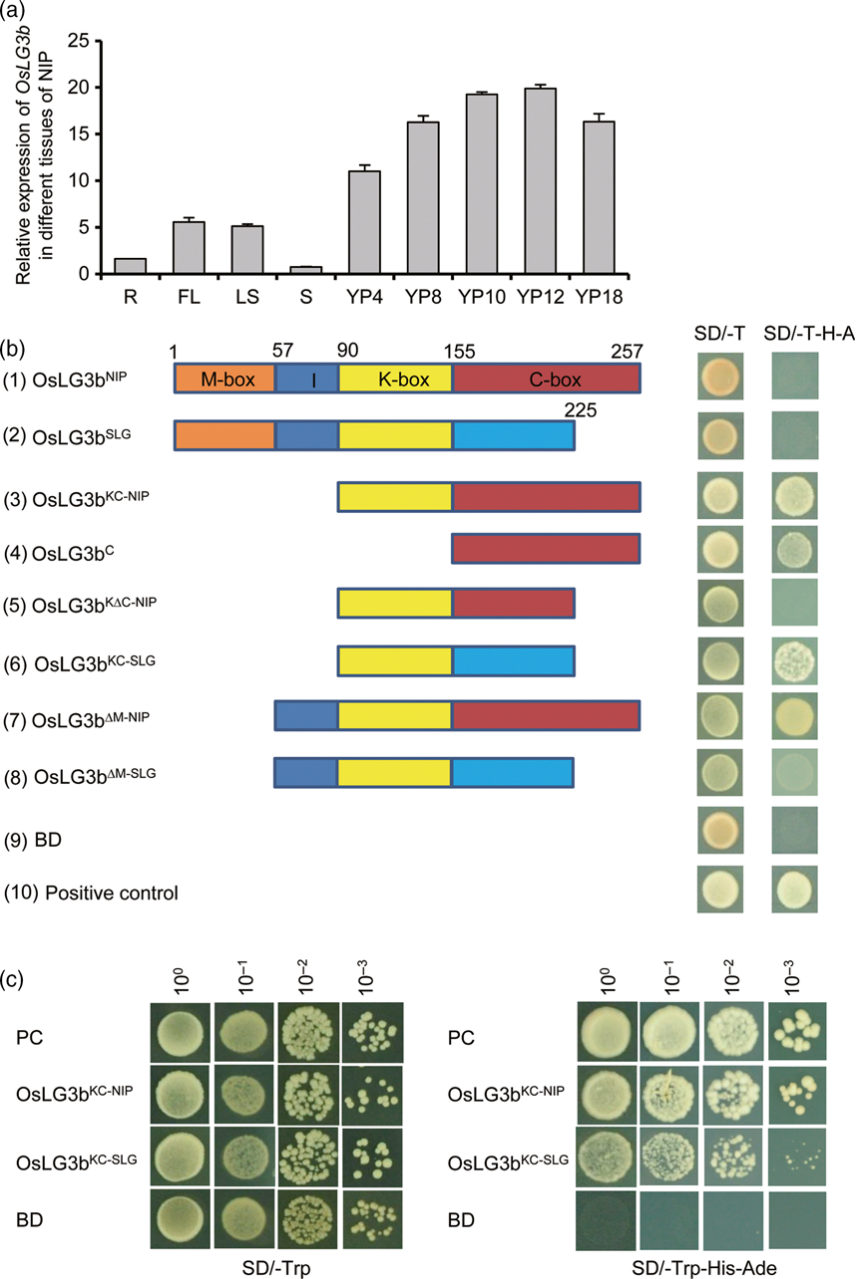
Expression pattern of *OsLG3b* and its transcription activator activity. (a) Quantitative RT‐PCR analysis of *OsLG3b* in different tissues of NIP. R, root; FL, flag leaf; LS, leaf sheath; S, stem; YP4, YP8, YP10, YP12 and YP18 represent young panicles with average lengths of 4, 8, 10, 12 and 18 cm, respectively. *n* = 3. Data are given as mean ± S.E.M. (b) Determination of the transcriptional activation domain in OsLG3b. The full‐length cDNA of OsLG3b and DNA fragments responsible for different truncated deletions from NIP and SLG were introduced into the pGBKT7 vector. BD represents GAL4 DNA binding domain. The empty pGBKT7 was the negative control. The numbers on the top of the panel indicate amino acid positions in the wild‐type *OsLG3b* gene. Brown, blue, yellow and red boxes depict the MADS, I, K, and C domains, respectively. (c) Transcription activity assay between OsLG3b ^KC^ ^‐^ ^NIP^ and OsLG3b ^KC^ ^‐^ ^SLG^ . Serial dilutions of 10^0^–10^−3^ transformed yeast cells were grown on SD‐Trp/‐His/‐Ade medium.

We searched the Rice Expression Profile Database (RiceXPro), the HANADB‐Os database and the eFP browser of rice microarray data (Arora *et al*., 2007; Hanada *et al*., 2007; Hruz *et al*., 2008) for *OsLG3b* expression data. Expression levels of *OsLG3b* in the inflorescence, lemma, palea, pistil and ovary were higher than in other tissues and organs at various developmental stages (Figure S9a, b). These results indicated that *OsLG3b* expression was higher during panicle and seed development than at other stages (Figure S9c, d). The gene was highly expressed in glume tissues and weakly expressed in other organs (Figure S9d). We also conducted an expression analysis of *OsLG3b* in Nipponbare. The results showed a higher transcriptional level of *OsLG3b* in developing panicles (Figure 3a). The results were consistent with previous conclusions that *OsLG3b* was involved in specifying the identities of lemma and palea (Khanday and Vijayraghavan, 2013; Prasad *et al*., 2001).

OsLG3b/OsMADS1 is a MIKC^C^‐type MADS‐box transcription factor containing four domains (MADS‐box, I region, K‐box and C‐box) (Arora *et al*., 2007; Jeon *et al*., 2000). The activity of a series of truncations of OsLG3b was examined, and the results showed that the C‐box region of OsLG3b was sufficient to activate the reporter (Figure 3b). This indicated that the C‐terminal region of OsLG3b had transcriptional activation activity and that the N‐terminal region containing the MADS‐activabox had DNA binding activity, consistent with previous reports (Lim *et al*., 2000). However, the full‐length OsLG3b from both SLG and Nipponbare showed abolished transcriptional activation function in yeast cells, and mutants with deletions of the MADS domain from Nipponbare exhibited strong transcriptional tor functionality (Figure 3b, (1), (2) and (7)). That is, the transactivation domain of OsLG3b is presumably inhibited in its full‐length native form, implying a unique conformational regulation of the transcriptional activation domain (Qiao *et al*., 2016; Zhang *et al*., 2015). Although mutants with deletions of the C‐terminal end of the C domain (amino acids 225–257; Figure 3b, (5)) had no transcriptional activity, the C domain of SLG OsLG3b showed weaker transcriptional activity than that of Nipponbare OsLG3b (Figure 3b, (3) and (6) and Figure 3c). Thus, alternative splicing of OsLG3b influences the transcriptional activation capacity of the OsLG3b protein.

### Targeted gene mutation of *OsLG3b* using a CRISPR‐Cas9 system

To further investigate the function of *OsLG3b*, we conducted targeted gene mutation of *OsLG3b* in rice using a CRISPR‐Cas9 system. Positive transgenic plants (T_1_ generation) were named RM23, RM25 and RM29 (Figure S10a‐c, h). The target sequence (5′‐AGATCAGGGTGACCATTCCC‐3′) was at sites +7569–+7570 within the seventh exon that encoded the C‐terminal of OsLG3b (amino acid residues 220–227). Sequencing analyses identified 2‐bp deletions (TC7568‐P7569, CA7569‐7570 and CA7569‐7570) in knockout lines. These deletions caused the frameshift mutation that resulted in differences in the C‐terminal of OsLG3b and consequent incomplete polypeptide that lacked OsLG3b function. Transgenic plants at the vegetative stage showed no visible differences from Nipponbare (Figure S10i). However, panicles of transgenic plants exhibited phenotypic differences, including spikelets consisting of elongated leafy paleae and lemmas with open hulls, and sharply decrease fertility (Figure S10d‐g). These results demonstrated that *OsLG3b* played an important role in floral organ development in rice, particularly in floral glume development (Prasad *et al*., 2005).

### Hap‐SLG of *OsLG3b/OsMADS1* appeared during tropical *japonica* improvement

**Figure 4:**
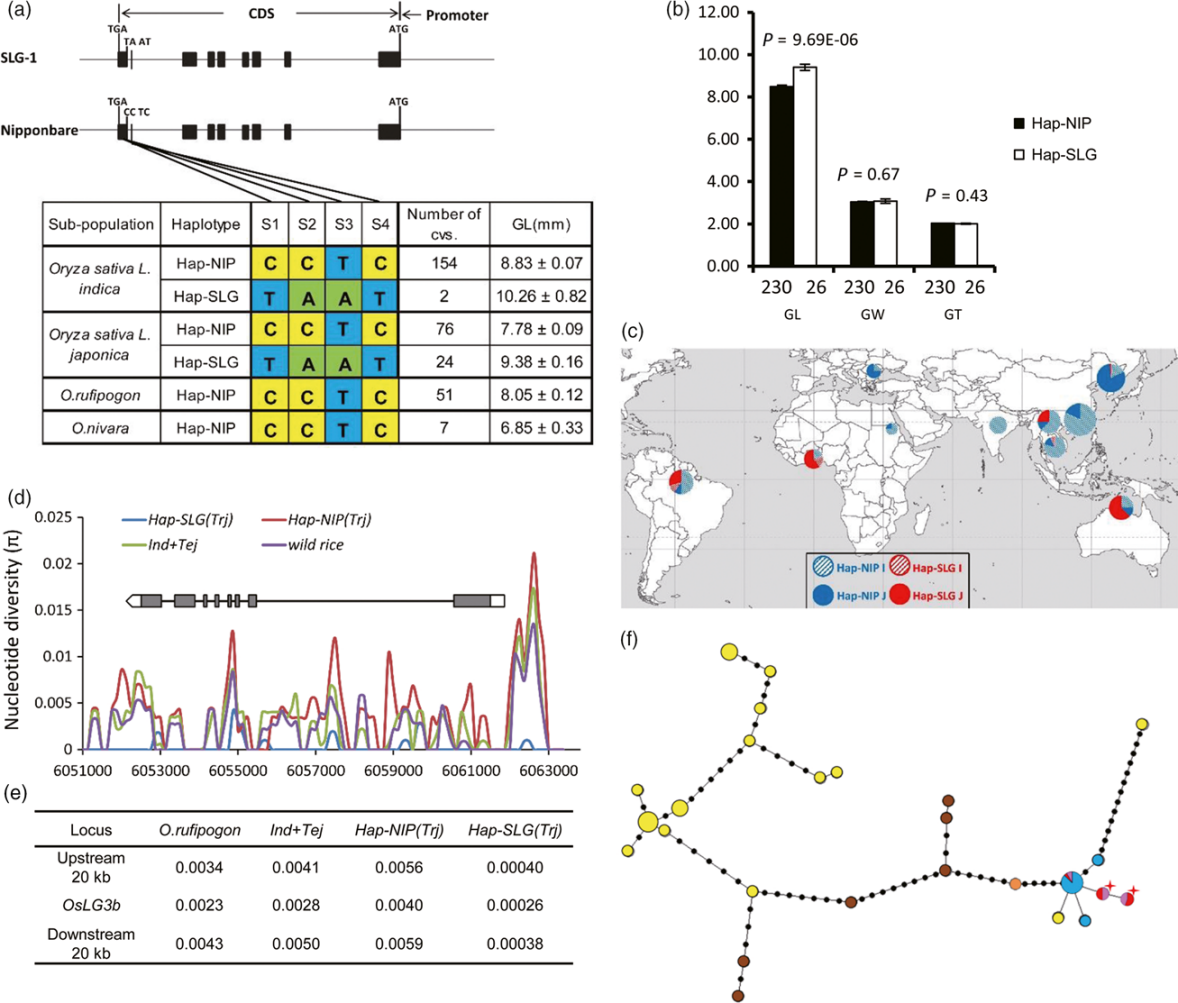
The haplotypes and origin of *OsLG3b*. (a) Haplotype analysis of *OsLG3b*. Gene structure and natural variation between alleles from SLG and NIP (top). Natural variation in *OsLG3b* among 256 rice accessions on a mini‐core collection compared with the NILs (bottom). (b) Grain shape of accessions representing the two classes in MCC1. GL,grain length; GW, grain width; GT, grain thickness. Raw data are provided in Table S1; *n*, is the number of accessions. *P*‐values were generated by two‐tailed *t*‐tests. Error bars, S.E.M. (c) Geographic origin of accessions containing the *OsLG3b* ^*SLG*^ allele. Hap‐NIP and Hap‐SLG are represented by red and blue circles, respectively. *Indica* and *japonica* cultivars are denoted by dashed and solid circles, respectively. (d) Nucleotide diversity analysis in the *OsLG3b* region. Representative samples included 35 *indica* accessions, 30 temperate *japonica* accessions, 21 tropical *japonica* accessions and 10 wild rice accessions (Table S5). Tej, temperate *japonica*; trj, tropical *japonica*. *Y*‐axis, average π value; *x*‐axis, Nipponbare TIGR v7.0 genome coordinate of chromosome 3. (e) Average nucleotide diversity of the 20 kb surrounding *OsLG3b*. (f) A minimum‐spanning tree for the *OsLG3b* region including 504 diverse rice sequences and 10 diverse wild rice sequences. Each haplotype group is represented by a circle, and circle size represents the number of lines within the haplotype, as in Figure 4c. Orange, brown, yellow, red, blue and pink represent *O. rufipogon* from China, *O. rufipogon* from South‐East Asia, *indica*, tropical *japonica*, temperate *japonica* and temperate *japonica* with glutinous varieties from the Yunnan–Guizhou Plateau or mixtures with recently improved *indica* or tropical *japonica*. Red star corresponds to *OsLG3b* ^*SLG*^ .

On the basis of the four most significant SNPs (*P* < 1.0 × 10^−12^) identified by association mapping (Figure 2a), we divided the sequences from the cultivated varieties in the MCC (Table S5) into two haplotypes (Figure 4a) and determined whether accessions in the *indica* group and *japonica* groups with the haplotype Hap‐SLG showed longer grains than those with Hap‐NIP (Figure 4a, b).

To understand the origins of the alleles, we sequenced the *OsLG3b* gene in 58 wild rice accessions, including 7 *O. nivara* and 51 *O. rufipogon* (Table S5). All wild accessions carried Hap‐NIP at the *OsLG3b* locus. From wild rice to *O. sativa*, almost all *indica* accessions and temperate *japonica* accessions had Hap‐NIP. Geographically, *japonica* accessions are mainly distributed in northern regions, whereas *indica* accessions are planted mainly in southern areas. Fourteen tropical *japonica* cultivars and two *indica* accessions with Hap‐SLG were distributed in tropical areas, such as Australia, Brazil, Ivory Coast, Nigeria and Indonesia. Another nine *japonica* samples which also had Hap‐SLG type were distributed on the Yunnan–Guizhou Plateau at higher elevations of the south‐east zone of Asia (Figure 4c). Within tropical *japonica*, four of five landrace accessions and four improved varieties were also Hap1 type, whereas up to 14 improved varieties contained Hap‐SLG type (Figure S11). Interestingly, a few recently improved *indica* and *temperate japonica* accessions also contained Hap‐SLG type. We produced a phylogenetic tree of the MCC population by cluster analysis using the STRUCTURE algorithm and found genomic segments of tropical *japonica* in those varieties (Table S5). These results suggest that Hap‐SLG type arises after domestication of tropical *japonica* and then spread to other group in breeding practice.

We performed an analysis of nucleotide diversity for the *OsLG3b* region from 96 accessions (Table S5). A comparative analysis of the nucleotide diversity among wild rice, *O. sativa* (*indica* and temperate *japonica*), Hap‐NIP(*Trj*) and Hap‐SLG(*Trj*) accessions indicated that on average, Hap‐SLG(*Trj*) accessions exhibited much lower diversity (π = 0.00026) than Hap‐NIP(*Trj*) (π = 0.0040), *O. sativa* (π = 0.0028) and wild rice species (π = 0.0023) (Figure 4d). Significantly decreased diversity in Hap‐SLG(*TRJ*) cultivars could be a result of artificial selection (Nielsen *et al*., 2007; Oleksyk *et al*., 2010). In addition, we calculated the nucleotide diversity of the *OsLG3b* flanking regions. As expected, we found that the average nucleotide diversity in 20‐kb flanking regions in Hap‐SLG(*Trj*) cultivars (π = 0.0004) was much lower than those of all other groups (π = 0.0057 for Hap‐NIP(*Trj*); π = 0.0020 for *O. sativa*; π = 0.0039 for wild rice) (Figure 4e). These observations indicate that the *OsLG3b* allele conferring long grains is selected during improvement of tropical *japonica* rice.

To further investigate the evolution of *OsLG3b*, we increased the size of population for next analysis. Nineteen haplotypes from 504 cultivated rice (14 *indica* and 5 *japonica* haplotypes) and seven haplotypes from 15 wild rice accessions were identified. Two distinct groups, namely a cultivar haplotype cluster and a wild rice haplotype cluster were shown in a minimum‐spanning tree of these *OsLG3b* haplotypes (Figure 4f). Wild rice demonstrated haplotypes with small grain. Several Chinese *Oryza rufipogon* rice showed similar haplotypes to Hap‐NIP and these might be inherited to ancient *japonica* (Huang *et al*., 2012b). Almost all *indica* and *temperate japonica* haplotypes had the small grain allele of *OsLG3b*, indicating that the large‐grain haplotypes in tropical *japonica* might have occurred after domestication and increased in frequency during adaptation of *japonica* to tropical regions.

### Effect of four‐gene combinations on grain length in natural rice varieties

**Figure 5:**
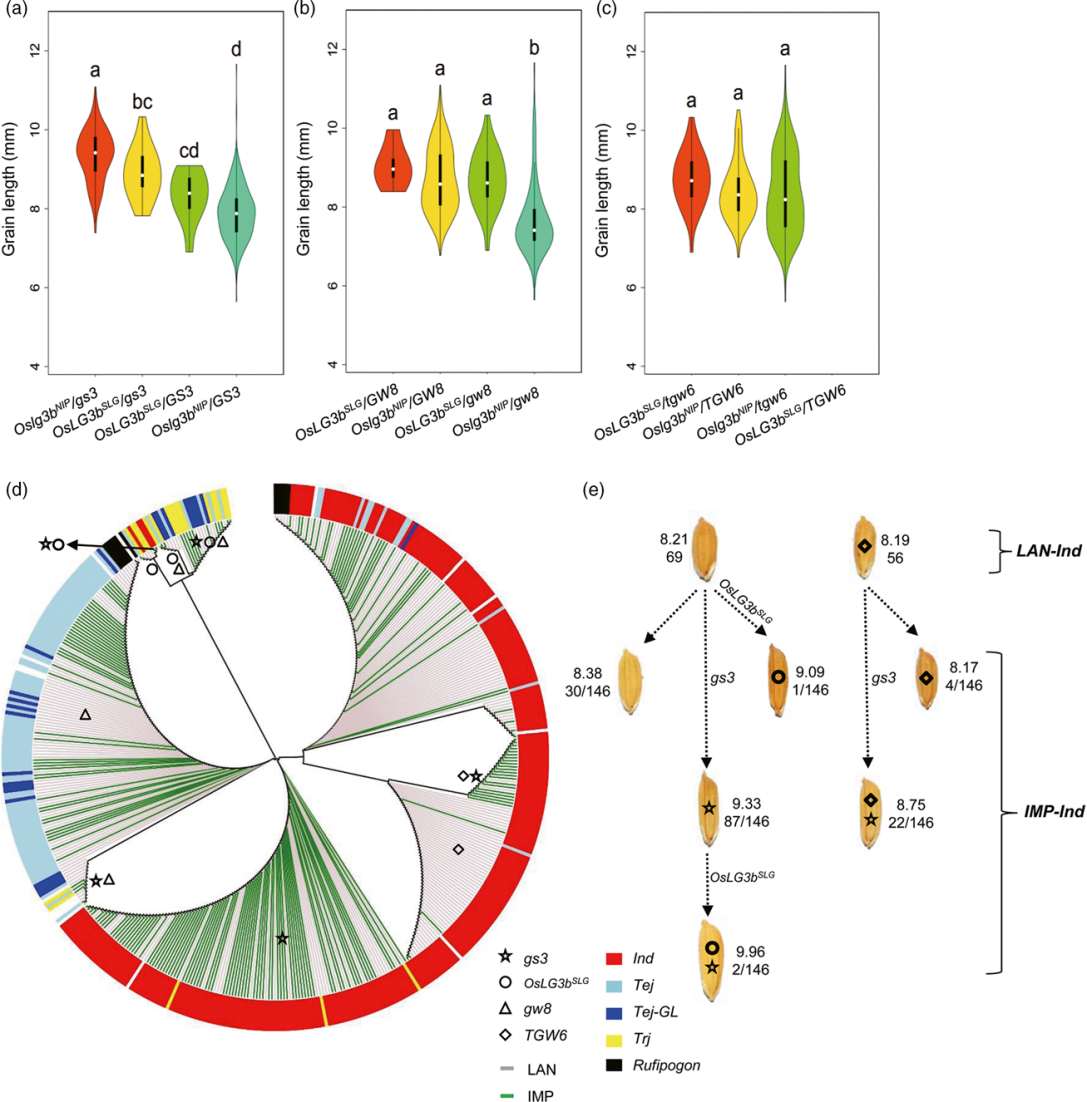
Genetic interactions between *OsLG3b* and other grain length‐related genes based on diverse germplasm and breeding improvement of *OsLG3b*. Box plot and kernel density plots were generated as violin plots for different groups. (a–c) Relationship between *OsLG3b* and other grain size–related genes, including *GS3* (a), *GW8* (b), and *TGW6* (c). *OsLG3b* ^*SLG*^ and *Oslg3b* ^*NIP*^ refer to the SLG allele and NIP allele of *OsLG3b*, respectively. The violin map was constructed in R. Different letters above columns indicate statistically significant differences between groups (Tukey's honestly significant difference (HSD) test, *P* < 0.05). Landraces, genotypes and phenotypes are listed in Table S5. (d) Phylogenetic analysis of four grain length‐related genes in 480 accessions. The phylogenetic tree of 480 varieties was constructed based on different functional SNPs or indels (listed in Table S5) by MEGA 6.0. All varieties were categorized by allelic variations in the FNP of *OsLG3b, GS3, GW8* and *TGW6*. The small pentagram, circle, triangle and prism refer to the beneficial alleles of *gs3*, *OsLG3b*, *GW8* and *TGW6*, respectively. Gainsboro lines refer to landrace varieties and green lines to improved varieties. Different colours reflect the different subgroups, with abbreviations as in Figure 4. (e) Improvement of haplotypes combinations of four grain length‐related genes, as in Figure 5d. Top numbers indicate average grain length; bottom numbers correspond to accession number with a haplotype combination in that subgroup.

We further examined haplotypes of *OsLG3b*, *GS3, GW8* and *TGW6* in 480 accessions to understand the genetic interaction between *OsLG3b* and other genes controlling grain length. We classified all varieties by the functional SNP (FNP) of *OsLG3b*, *GS3, GW8* and *TGW6*. As shown in Figure 5a‐b, in the presence of the *OsLG3b* ^*SLG*^ allele, no significant difference was observed in the grain length between the groups containing the *GS3/GW8* and *gs3/gw8* alleles, respectively. This implied that the *OsLG3b* ^*SLG*^ allele had an epistatic effect on grain length for *GS3* and *GW8*. Nevertheless, there was no evidence of interaction between *OsLG3b* and *TGW6* (Figure 5c). These results showed that some of these genes might synergistically determine grain length (Table S5). As shown in Figure 5d, landraces and breeding varieties in *indica* were clearly distinguished due to improved grain length, however, that mixed together well in *japonica* (Figure 5d). This implied that grain length was widely meliorated in *indica*, whereas there was considerable scope for improving grain length in temperate *japonica*. However, in the *indica* and temperate *japonica* group, a few improved varieties had significantly longer grain that might be attributable to introgression of the beneficial *OsLG3b* allele (Figure 5d, e), suggesting that *OsLG3b* might be potential resource for improving grain length in rice breeding.

### Introgression of the tropical *japonica* allele of *OsLG3b* into *indica* and temperate *japonica*

**Figure 6:**
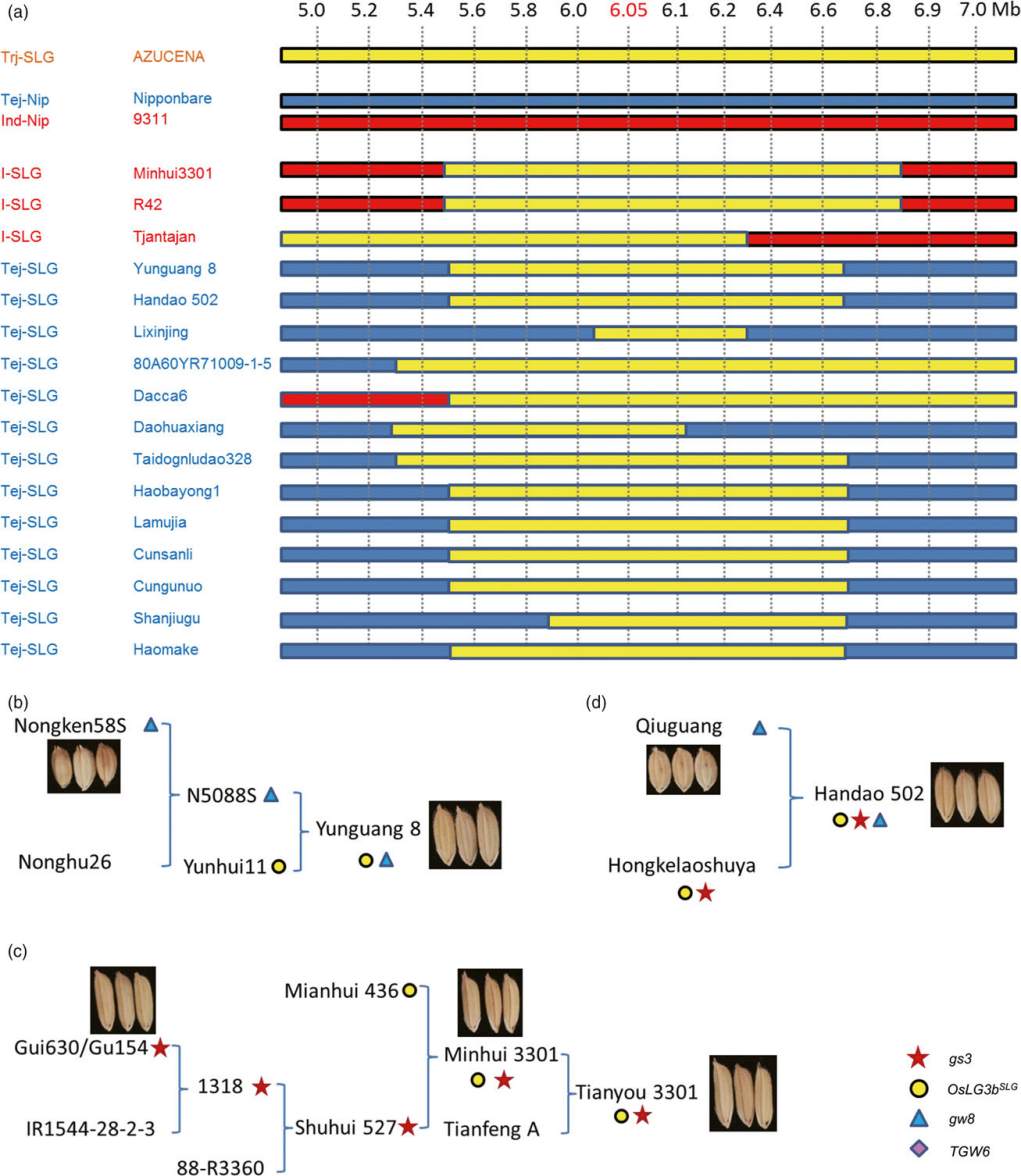
Analysis of introgressed regions and application of *OsLG3b* in breeding. (a) Introgression of the tropical *japonica* allele of *OsLG3b* into *indica* and temperate *japonica*. Fourteen sequential markers in the 5.0‐ to 7.0‐Mb region of chromosome 3 were constructed at approximately 0.2‐Mb intervals. Three *indica* and thirteen temperate *japonica* lines with the SLG‐type *OsLG3b* were sequenced at these markers with AZUCENA (representative tropical *japonica*), ‘Nipponbare’ (temperate *japonica*) and 93‐11 (*indica*) as reference sequences. Yellow, blue and red bars indicate tropical *japonica,* temperate *japonica* and *indica* type genotypes, respectively. Trj/Tej/I‐SLG, tropical/temperate *japonica*/indica accessions with the SLG‐type *OsLG3b*; Tej/I‐Nip, temperate *japonica*/*indica* accessions with the Nipponbare‐type *OsLG3b*. The marker at 6.05 Mb was the closest to *OsLG3b*. (b‐d) The pedigree of selected rice varieties. (b) Yunguang 8, (c) Tianyou 3301 and (d) Handao 502. The red star, yellow dot, blue triangle and purple prism refer the beneficial allele of *gs3*, *OsLG3b*, *GW8* and *TGW6,* respectively.

We found that a few *indica* and temperate *japonica* varieties had the same large‐grain haplotype of *OsLG3b* as tropical *japonica* (Table S5). To determine if these accessions were the result of introgression across varietal groups, we conducted an analysis of introgressed regions. We chose Nipponbare (the reference temperate *japonica*), 9311 (the reference *indica*) and AZUCENA (a typical tropical *japonica* landrace) as reference sequences for subsequent genotyping analyses. Three *indica* and thirteen temperate *japonica* lines with the *OsLG3b* ^*SLG*^ allele were then subjected to genotyping based on fourteen markers in the 5.0‐ to 7.0‐Mb region of chromosome 3. We found that all of them had a small chromosomal segment identical to that in the genomes of the tropical *japonica*. This implied that genomic regions (minimum length of ~300 kb) containing the *OsLG3b* ^*SLG*^ gene from tropical *japonica* had been introgressed into the genome of *indica* and temperate *japonica* varieties (Figure 6a). Therefore, the *OsLG3b* ^*SLG*^ allele might have been transferred from tropical *japonica* to *indica* or temperate *japonica* by introgression. To further clarify these events, 39 SNPs in the proximal region of the *OsLG3b* gene were examined. Although the accessions belonged to the *indica* and temperate *japonica* groups, the SNP pattern around *OsLG3b* was similar to the tropical *japonica* type (Tables S5 and S10). We also manually examined the patterns of 39 SNPs in representative wild rice accessions (Xu *et al*., 2012), and we found that eight of them were more similar to the *indica*, whereas others were more similar to the temperate *japonica* (Table S10). All were less similar to the tropical *japonica* than to *indica* and temperate *japonica* rice (Figure S13). These results showed that the *OsLG3b* ^*SLG*^ allele originated in a tropical *japonica* line and spread to *indica* and temperate *japonica* by natural crossing and artificial selection.

## Discussion

### Alternative splicing of *OsLG3b/OsMADS1* arose during improvement of tropical *japonica*

We show that an alternative splicing of *OsLG3b* endows tropical *japonica* rice with large grain and that a mutation in the coding region of *OsLG3b* was fixed in tropical *japonica* cultivars. *O. rufipogon*, a type of wild rice, is recognized as the direct progenitor of cultivated rice based on a comprehensive data set obtained from genomic sequences of 446 geographically diverse wild rice accessions and 1,083 cultivated *indica* and *japonica* varieties (Huang *et al*., 2012b). However, our evolutionary analyses based on abundant SNPs from cultivated and wild rice further demonstrated that the alternative splicing allele might have arisen after domestication. Further introgression analysis showed that the alternative splicing allele originated in the tropical *japonica* and was subject to strong human selection during improvement of tropical *japonica* (Tajima *D* = −1.80, *P* < 0.05; MLHKA = 0.007), similar to the events relating to the *GS3* gene for tropical *japonica* improvement (Takano‐Kai *et al*., 2009).

### *OsLG3b* encodes a MIKC^C^‐type MADS‐box transcription factor involved in regulation of grain length and weight

MIKC^C^‐type MADS‐box genes play a very important role in plant growth and development, such as control of flowering time, regulation of root, leaf, ovule and fruit development, and especially determination of floral meristem and floral organ traits (Ferrario *et al*., 2006; Komiya *et al*., 2009; Lee *et al*., 2008; Mara and Irish, 2008; Moyle *et al*., 2005; Prasad *et al*., 2001, 2005; Zhang *et al*., 2008). There are 38 MIKC^C^‐type genes in rice, of which *OsMADS1*, *OsMADS5*, *OsMADS7*, *OsMADS8* and *OsMADS34* belong to the E class. Prasad *et al*. (2005) showed that *OsMADS1* was an early‐stage regulatory factor of inner floral organs, and controlled differentiation of specific cell types in the lemma and palea. In a loss‐of‐function mutant of *OsMADS1*, the glume, stamens and carpels were transformed into hull or glume structures, and the determinacy of the floral meristem was lost (Agrawal *et al*., 2005; Jeon *et al*., 2000; Khanday and Vijayraghavan, 2013; Liu *et al*., 2016; Prasad *et al*., 2005; Wang *et al*., 2010). In this study, *OsLG3b* was cloned by a forward genetics method, and functional natural variation was identified using broad germplasm resources. *OsLG3b* was annotated as a MIKC^C^‐type MADS‐box transcription factor 1. Sequence analysis revealed that nucleotide substitutions in exon 8 resulted in alternative splicing and a truncated protein at the C terminus, which was retained in domestication of tropical *japonica*. These natural variations do not occur in the MADS‐box and K‐box conserved domains where mutation would likely lead to severe defective phenotypes. The natural variations resulted in increased grain length and grain thickness with potential value in breeding for improved yield.

### Molecular improvement of grain length and weight in rice

Grain size has played an important part in the evolution and improvement of rice (Kovach *et al*., 2007). Breeders have made great progress in rice improvement and created abundant accessions with different grain lengths. It might be meaningful to lift the veil of molecular imprinting that traces to preference from artificial selection. Distinct genes have been selected in two subpopulations for improving grain length and weight (Figure 5d). For this, we had note that some of the influences that worked on the selection might have come from the geographical distribution pattern, time of occurrence of mutation, preference of local people and effect of the gene itself. For example, the mutant allele of *GS3* had been proved to be origin from tropical *japonica* (Wang *et al*., 2011) and arose during improvement of *indica* varieties. This allele presumably resulted from introgression from either tropical *japonica* or *indica* by natural and artificial crossing (Figure 5d and Table S5). The mutant allele of *GS3* had been widely applied in low latitude region because of its visual effect on grain length; however, it was rare in the northern temperate *japonica* subgroup, perhaps a result of the combined influence of the above‐mentioned four influences (Figure S14a, b and Table S5).

It is interesting that *GW8* and *TGW6* seemingly could not alter grain length in our germplasm resources on account of a strong correlation between the distribution of their two alleles and population structure (Figure S14c‐f). This distribution pattern might be the reason that breeders did not recognize and utilize these genes. Whether in the temperate *japonica* or *indica* subgroups, from landraces to improved varieties, the *GW8* allele was unchanged (Figure 5d). In our MCC panel, the beneficial allele of *TGW6* only existed in a very small number of *indica* landraces, many of them with inferior yield characteristics, and the phenotypic effect on grain length was so small that it was not selected and used by breeders. Our findings suggest that there is still much potential for optimizing grain shape by marker‐assisted selection.

### Application of *OsLG3b* in breeding

It is very difficult for rice breeders to improve yield and quality at the same time, because frequently grain yield is negatively correlated to grain quality. For example, although *GW2*, *GS2*, *GW5* and *GW8* increase rice production, they lead to bad quality. NIL(SLG) offers superior grain weight and grain yield. Here, we evaluated whether the SLG *OsLG3b* allele caused poor grain quality. Our results revealed that there was no significant difference between NIP and NIL(SLG) in percentage and degree of chalkiness. Both the NIP and NIL(SLG) endosperms comprised largely sharp‐edged and compactly arranged polygonal starch granules, which are quite often associated with a low level of grain chalkiness (Figure S15a‐j). The findings showed that *OsLG3b* effectively increased yield in rice, but did not affect quality.

The beneficial allele of *OsLG3b* likely spread into some *indica* and temperate *japonica* varieties by repeated introgressions, probably through natural crosses and artificial selection (Figure 6a and Figure S14g, h). Yunguang 8 is an elite variety widely grown in Yunnan province. According to the pedigree records, its parents are the Yunhui11 and Nongken58S (Figure 6b). A resequencing study showed that Yunguang 8 carried the *OsLG3b* ^*SLG*^ and the *gw8* ^*Basmati*^ allele (Figure 6b). Thus, combination of the *OsLG3b* ^*SLG*^ and *gw8* ^*Basmati*^ alleles provides a good example that can be followed by breeders to simultaneously improve yield and grain quality over current levels. Moreover, Tianyou 3301, a well‐known super rice, represents another practical example of combining the *OsLG3b* ^*SLG*^ and *gs3* alleles and alleles for other unidentified yield‐related genes. There are also many other breeding examples (e.g. Handao502, an elite upland rice) (Figure 6d). Our results strongly indicate that the route of varietal improvement will be revealed by molecular imprinting. In turn, knowledge of molecular imprinting of grain and yield‐related alleles might help in breeding rice varieties with high yield and superior quality.

## Experimental procedures

### Plant materials and growing conditions

SLG‐1 (*ssp*. *japonica*) was screened from more than 7000 accessions and used as a donor of desirable alleles. Nipponbare (*ssp. japonica*) was selected as a recurrent parent. SLG was crossed with NIP to produce F_1_ plants, and that were subsequently backcrossed with NIP to produce 30 BC_3_F_1_ plants and 73 BC_4_F_1_ plants. Peak values for grain weight in BC_3_F_1_ plants were 27 g and 31 g, whereas there were four peak values among BC_4_F_1_ plants, 28 g, 30 g, 33 g and 38 g. The four BC_4_F_1_ plants at peak value were selfed to produce four BC_4_F_2_ populations for primary QTL mapping. Three BC_4_F_3_ populations for further QTL mapping were derived from BC_4_F_2_ plants lacking other large‐effect QTLs. Plant BC_4_F_3_‐78‐11 was crossed with NIP and then selfed to produce a BC_5_F_2_ population for fine mapping. All plant materials were grown under natural field conditions as described previously (Yu *et al*., 2017).

### QTL mapping and fine mapping

MapMaker3.0 and IciMapping3.1 were used for genetic map construction and QTL analysis, respectively (Li *et al*., 2007). Composite interval mapping was applied with the LOD threshold 2.5. Fine mapping of *qGL3‐2* was based on 6000 BC_5_F_2_ plants and 4100 BC_5_F_2_ plants. The relevant primer sequences are listed in Table S4 and S11.

### Candidate region association mapping

The mini‐core collection panel was collected from 35 countries and had abundant diversity. A generalized linear model (GLM) method, taking account of population structure (Q), was used to perform regional association mapping. The Q matrix was estimated by STRUCTURE 2.0. Statistical analyses for LD and association were carried out using TASSEL, version 4.0.

### Vector construction and rice transformation

To generate the overexpression vector, the ORF of OsLG3b was amplified from the cDNA of SLG and cloned into the pMDC32 vector. sgRNA‐Cas9 plant expression vectors were constructed as described previously (Mao *et al*., 2013). The primers used in vector construction are listed in Table S12. Two constructs were transformed to generate transgenic plants by *Agrobacterium*‐mediated transformation (Yu *et al*., 2017).

### Transactivation activity assay

The cDNA of *OsLG3b* and DNA fragments responsible for different truncated deletions from NIP and SLG was introduced into the pGBKT7 vector. The vectors were then transformed into yeast strain AH109 and screened as described elsewhere (Yu *et al*., 2017). Relevant primer sequences are listed in Table S12.

### Evolution analysis of *OsLG3b*

To investigate the domestication and improvement of *OsLG3b*, we conducted some population genetic analyses. A Perl script was employed to calculate the sequence diversity. Construction of a minimum‐spanning tree followed a described procedure (Liu *et al*., 2015). The neighbour‐joining tree was drawn by MEGA 6.0, and the number of bootstrap replicates was 1000 times. Presentation of the phylogenetic tree was guided by EvolView (Zhang *et al*., 2012a). Relevant landraces, phenotypes and polymorphism data are given in Table S5.

### URLs

Gene annotation referred to RGAP (http://rice.plantbiology.msu.edu/). All SNP data were acquired from the Rice Functional Genomics and Breeding database (RFGB, http://www.rmbreeding.cn/Index/).

## Competing interests

The authors declare that they have no competing interests.

## Supporting information


**Figure S1** (a) Grains from 10 typical *tropical japonica* varieties (CH1027, CH1029, CH1067, CH1091, CH1083, CH1085, CH1086, CH1058, IRAT109 and Haogelao), and 10 typical *temperate japonica* varieties (CH1001, CH1002, CH1004, CH1008, CH1009, CH1010, CH1020, CH1026, CH1071, and Nipponbare) (Table S5).
**Figure S2** Graphic genotype of BC_4_F_3_‐78‐11.
**Figure S3** Field trial of NIP and NIL(SLG) plants.
**Figure S4** Geographic origins of 266 *indica* and *japonica* rice accessions.
**Figure S5** Frequency distribution of grain length in the mini core collection (MCC population) (Yu *et al*., 2017).
**Figure S6** Amino acid sequence alignment of *OsLG3b* from Nipponbare (Nip), SLG and IRAT109.
**Figure S7** Grains from SLG, Nipponbare, and IRAT109. Scale bar, 5 mm.
**Figure S8** cDNA sequence alignment of *OsLG3b* from Nipponbare (Nip), SLG and IRAT109.
**Figure S9** The temporal‐spatial expression pattern of *OsLG3b*.
**Figure S10** Phenotypic analysis of CRISPR*‐OsLG3b* transgenic plants.
**Figure S11** Genotypes of *OsLG3b* in tropical *japonica* and *indica* or temperate *japonica* admixed with tropical *japonica* between landraces and improved varieties.
**Figure S12** Histograms showing distribution of grain length, grain width, length: width ratio and grain weight in temperate *japonica* (*Tej*) and tropical *japonica* (*Trj*) accessions.
**Figure S13** Phylogenetic tree of the representative wild rice accessions and sixteen *indica* or temperate *japonica* lines with the *OsLG3b* ^*SLG*^ allele.
**Figure S14** Comparison of grain lengths in large‐grain and small‐grain haplotypes for *GS3* (a), *GW8* (c), *TGW6* (e) and *OsLG3b* (g) when Q structure (sub1, *indica*; sub2, *japonica*) exists.
**Figure S15** *OsLG3b* does not affect grain quality.
**Table S1** Means differences for the selected grain traits identified with *t* tests between *temperate japonica* and *tropical japonica*.
**Table S2** Identification of QTLs related to grain length, grain width, grain thickness and grain weight.
**Table S3** Analysis of polymorphisms at function variations’ sites between Nipponbare and SLG.
**Table S6** Summary of the taxa and source of 506 varieties.
**Table S7** Environments used to evaluate association and linkage populations.
**Table S8** The heritability of grain traits in MCC Panel.
**Table S9** *OsLG3b* polymorphisms associated with grain length in the MCC panel.

**Table S4** Primers used for the genotyping of a near‐isogenic line for the *qGL3‐2* locus.

**Table S5** Information of *Orayza sativa* L. varieties and wild rice on variety name, geographic source, stratification referred by STRUCTURE, the integrated stratification, grain lengths and allelic variations of grain‐length‐related genes.

**Table S10** Thirty‐nine SNPs in *OsLG3b* used in the introgression analysis.

**Table S11** Primers used for fine mapping and sequencing.

**Table S12** Primers used for DNA constructs and transcripts analysis.

## References

[pbi12903-bib-0001] Agrawal, G.K. , Abe, K. , Yamazaki, M. , Miyao, A. and Hirochika, H. (2005) Conservation of the E‐function for floral organ identity in rice revealed by the analysis of tissue culture‐induced loss‐of‐function mutants of the *OsMADS1* gene. Plant Mol. Biol. 59, 125–135. 16217607 10.1007/s11103-005-2161-y

[pbi12903-bib-0002] Arora, R. , Agarwal, P. , Ray, S. , Singh, A.K. , Singh, V.P. , Tyagi, A.K. and Kapoor, S. (2007) MADS‐box gene family in rice: genome‐wide identification, organization and expression profiling during reproductive development and stress. BMC Genom. 8, 242. 10.1186/1471-2164-8-242 PMC1947970 17640358

[pbi12903-bib-0004] Duan, P. , Xu, J. , Zeng, D. , Zhang, B. , Geng, M. , Zhang, G. , Huang, K. *et al*. (2017) Natural variation in the promoter of *GSE5* contributes to grain size diversity in rice. Mol. Plant, 10, 685–694. 28366824 10.1016/j.molp.2017.03.009

[pbi12903-bib-0005] Fan, C. , Xing, Y. , Mao, H. , Lu, T. , Han, B. , Xu, C. , Li, X. *et al*. (2006) *GS3*, a major QTL for grain length and weight and minor QTL for grain width and thickness in rice, encodes a putative transmembrane protein. Theor. Appl. Genet. 112, 1164–1171. 16453132 10.1007/s00122-006-0218-1

[pbi12903-bib-0060] Fan, C. , Yu, S. , Wang, C. and Xing, Y. (2009) A causal C‐A mutation in the second exon of GS3 highly associated with rice grain length and validated as a functional marker. Theor. Appl. Genet. 118, 465–472. 19020856 10.1007/s00122-008-0913-1

[pbi12903-bib-0006] Ferrario, S. , Shchennikova, A.V. , Franken, J. , Immink, R.G. and Angenent, G.C. (2006) Control of floral meristem determinacy in petunia by MADS‐box transcription factors. Plant Physiol. 140, 890–898. 16428599 10.1104/pp.105.072660 PMC1400554

[pbi12903-bib-0007] Hanada, K. , Zhang, X. , Borevitz, J.O. , Li, W.H. and Shiu, S.H. (2007) A large number of novel coding small open reading frames in the intergenic regions of the Arabidopsis thaliana genome are transcribed and/or under purifying selection. Genome Res. 17, 632–640. 17395691 10.1101/gr.5836207 PMC1855179

[pbi12903-bib-0008] Hruz, T. , Laule, O. , Szabo, G. , Wessendorp, F. , Bleuler, S. , Oertle, L. , Widmayer, P. *et al*. (2008) Genevestigator v3: a reference expression database for the meta‐analysis of transcriptomes. Adv. Bioinform. 2008, 420747. 10.1155/2008/420747 PMC2777001 19956698

[pbi12903-bib-0009] Huang, R. , Jiang, L. , Zheng, J. , Wang, T. , Wang, H. , Huang, Y. and Hong, Z. (2012a) Genetic bases of rice grain shape: so many genes, so little known. Trends Plant Sci. 18, 218–226. 23218902 10.1016/j.tplants.2012.11.001

[pbi12903-bib-0010] Huang, X. , Kurata, N. , Wei, X. , Wang, Z. , Wang, A. , Zhao, Q. , Zhao, Y. *et al*. (2012b) A map of rice genome variation reveals the origin of cultivated rice. Nature, 490, 497–501. 23034647 10.1038/nature11532 PMC7518720

[pbi12903-bib-0011] Ishimaru, K. , Hirotsu, N. , Madoka, Y. , Murakami, N. , Hara, N. , Onodera, H. , Kashiwagi, T. *et al*. (2013) Loss of function of the IAA‐glucose hydrolase gene *TGW6* enhances rice grain weight and increases yield. Nat. Genet. 45, 707–711. 23583977 10.1038/ng.2612

[pbi12903-bib-0012] Jeon, J.‐S. , Jang, S. , Lee, S. , Nam, J. , Kim, C. , Lee, S.‐H. , Chung, Y.‐Y. *et al*. (2000) *leafy hull sterile1* is a homeotic mutation in a rice MADS box gene affecting rice flower development. Plant Cell, 12, 871–884. 10852934 10.1105/tpc.12.6.871 PMC149090

[pbi12903-bib-0013] Jin, J. , Hua, L. , Zhu, Z. , Tan, L. , Zhao, X. , Zhang, W. , Liu, F. *et al*. (2016) *GAD1* encodes a secreted peptide that regulates grain number, grain Length, and awn development in rice domestication. Plant Cell, 28, 2453–2463. 27634315 10.1105/tpc.16.00379 PMC5134979

[pbi12903-bib-0014] Khanday, I. and Vijayraghavan, U. (2013) Rice *LHS1/OsMADS1* controls floret meristem specification by coordinated regulation of transcription factors and hormone signaling pathways. Plant Physiol. 161, 1970–1983. 23449645 10.1104/pp.112.212423 PMC3613468

[pbi12903-bib-0015] Komiya, R. , Yokoi, S. and Shimamoto, K. (2009) A gene network for long‐day flowering activates *RFT1* encoding a mobile flowering signal in rice. Development, 136, 3443–3450. 19762423 10.1242/dev.040170

[pbi12903-bib-0016] Kovach, M.J. , Sweeney, M.T. and McCouch, S.R. (2007) New insights into the history of rice domestication. Trends Genet. 23, 578–587. 17963977 10.1016/j.tig.2007.08.012

[pbi12903-bib-0017] Lee, S. , Jeong, D.H. and An, G. (2008) A possible working mechanism for rice SVP‐group MADS‐box proteins as negative regulators of brassinosteroid responses. Plant Signal. Behav. 3, 471–474. 19704489 10.4161/psb.3.7.5677 PMC2634433

[pbi12903-bib-0019] Li, H. , Ye, G. and Wang, J. (2007) A modified algorithm for the improvement of composite interval mapping. Genetics, 175, 361. 17110476 10.1534/genetics.106.066811 PMC1775001

[pbi12903-bib-0020] Lim, J. , Moon, Y.H. , An, G. and Jang, S.K. (2000) Two rice MADS domain proteins interact with OsMADS1. Plant Mol. Biol. 44, 513–527. 11197326 10.1023/a:1026517111843

[pbi12903-bib-0021] Liu, H. , Liu, H. , Zhou, L. , Zhang, Z. , Zhang, X. , Wang, M. , Li, H. *et al*. (2015) Parallel domestication of the *heading date 1* gene in cereals. Mol. Biol. Evol. 32, 2726–2737. 26116860 10.1093/molbev/msv148

[pbi12903-bib-0022] Liu, M. , Li, H. , Su, Y. , Li, W. and Shi, C. (2016) G1/ELE functions in the development of rice lemmas in addition to determining identities of empty glumes. Front. Plant Sci. 7, 1006. 27462334 10.3389/fpls.2016.01006 PMC4941205

[pbi12903-bib-0023] Liu, J. , Chen, J. , Zheng, X. , Wu, F. , Lin, Q. , Heng, Y. , Tian, P. *et al*. (2017) *GW5* acts in the brassinosteroid signalling pathway to regulate grain width and weight in rice. Nat. Plants, 3, 17043. 28394310 10.1038/nplants.2017.43

[pbi12903-bib-0024] Mao, H. , Sun, S. , Yao, J. , Wang, C. , Yu, S. , Xu, C. , Li, X. *et al*. (2010) Linking differential domain functions of the GS3 protein to natural variation of grain size in rice. Proc. Natl. Acad. Sci. USA, 107, 19579–19584. 20974950 10.1073/pnas.1014419107 PMC2984220

[pbi12903-bib-0025] Mao, Y. , Zhang, H. , Xu, N. , Zhang, B. , Gou, F. and Zhu, J.K. (2013) Application of the CRISPR‐Cas system for efficient genome engineering in plants. Mol. Plant, 6, 2008. 23963532 10.1093/mp/sst121 PMC3916745

[pbi12903-bib-0026] Mara, C.D. and Irish, V.F. (2008) Two GATA transcription factors are downstream effectors of floral homeotic gene action in Arabidopsis. Plant Physiol. 147, 707–718. 18417639 10.1104/pp.107.115634 PMC2409029

[pbi12903-bib-0027] Moyle, R. , Fairbairn, D.J. , Ripi, J. , Crowe, M. and Botella, J.R. (2005) Developing pineapple fruit has a small transcriptome dominated by metallothionein. J. Exp. Bot. 56, 101–112. 15520025 10.1093/jxb/eri015

[pbi12903-bib-0028] Nielsen, R. , Hellmann, I. , Hubisz, M. , Bustamante, C. and Clark, A.G. (2007) Recent and ongoing selection in the human genome. Nat. Rev. Genet. 8, 857–868. 17943193 10.1038/nrg2187 PMC2933187

[pbi12903-bib-0029] Oleksyk, T.K. , Smith, M.W. and O'Brien, S.J. (2010) Genome‐wide scans for footprints of natural selection. Philos. Trans. R. Soc. Lond. B. Biol. Sci. 365, 185–205. 20008396 10.1098/rstb.2009.0219 PMC2842710

[pbi12903-bib-0030] Prasad, K. , Sriram, P. , Kumar, S.C. , Kushalappa, K. and Vijayraghavan, U. (2001) Ectopic expression of rice OsMADS1 reveals a role in specifying the lemma and palea, grass floral organs analogous to sepals. Dev. Genes. Evol. 211, 281–290. 11466523 10.1007/s004270100153

[pbi12903-bib-0031] Prasad, K. , Parameswaran, S. and Vijayraghavan, U. (2005) OsMADS1, a rice MADS‐box factor, controls differentiation of specific cell types in the lemma and palea and is an early‐acting regulator of inner floral organs. Plant J. 43, 915–928. 16146529 10.1111/j.1365-313X.2005.02504.x

[pbi12903-bib-0032] Qiao, Z. , Qi, W. , Qian, W. , Feng, Y.N. , Yang, Q. , Nan, Z. , Wang, S. *et al*. (2016) ZmMADS47 regulates zein gene transcription through interaction with Opaque2. PLoS Genet. 12, e1005991. 27077660 10.1371/journal.pgen.1005991 PMC4831773

[pbi12903-bib-0033] Shomura, A. , Izawa, T. , Ebana, K. , Ebitani, T. , Kanegae, H. , Konishi, S. and Yano, M. (2008) Deletion in a gene associated with grain size increased yields during rice domestication. Nature Genet. 40, 1023–1028. 18604208 10.1038/ng.169

[pbi12903-bib-0034] Song, X.J. , Huang, W. , Shi, M. , Zhu, M.Z. and Lin, H.X. (2007) A QTL for rice grain width and weight encodes a previously unknown RING‐type E3 ubiquitin ligase. Nature Genet. 39, 623–630. 17417637 10.1038/ng2014

[pbi12903-bib-0035] Takano‐Kai, N. , Jiang, H. , Kubo, T. , Sweeney, M. , Matsumoto, T. , Kanamori, H. , Padhukasahasram, B. *et al*. (2009) Evolutionary history of *GS3*, a gene conferring grain length in rice. Genetics, 182, 1323–1334. 19506305 10.1534/genetics.109.103002 PMC2728869

[pbi12903-bib-0036] Wang, K. , Tang, D. , Hong, L. , Xu, W. , Huang, J. , Li, M. , Gu, M. *et al*. (2010) *DEP* and *AFO* regulate reproductive habit in rice. PLoS Genet. 6, e1000818. 20107517 10.1371/journal.pgen.1000818 PMC2809758

[pbi12903-bib-0037] Wang, C. , Chen, S. and Yu, S. (2011) Functional markers developed from multiple loci in *GS3* for fine marker‐assisted selection of grain length in rice. Theor. Appl. Genet. 122, 905–913. 21107518 10.1007/s00122-010-1497-0

[pbi12903-bib-0038] Wang, S. , Wu, K. , Yuan, Q. , Liu, X. , Liu, Z. , Lin, X. , Zeng, R. *et al*. (2012) Control of grain size, shape and quality by *OsSPL16* in rice. Nature Genet. 44, 950–954. 22729225 10.1038/ng.2327

[pbi12903-bib-0039] Weng, J. , Gu, S. , Wan, X. , Gao, H. , Guo, T. , Su, N. , Lei, C. *et al*. (2008) Isolation and initial characterization of *GW5*, a major QTL associated with rice grain width and weight. Cell Res. 18, 1199–1209. 19015668 10.1038/cr.2008.307

[pbi12903-bib-0041] Xu, X. , Liu, X. , Ge, S. , Jensen, J.D. , Hu, F. , Li, X. , Dong, Y. *et al*. (2012) Resequencing 50 accessions of cultivated and wild rice yields markers for identifying agronomically important genes. Nature Biotechnol. 30, 105–111. 10.1038/nbt.2050 22158310

[pbi12903-bib-0042] Yu, J. , Xiong, H. , Zhu, X. , Zhang, H. , Li, H. , Miao, J. , Wang, W. *et al*. (2017) *OsLG3* contributing to rice grain length and yield was mined by Ho‐LAMap. BMC Biol. 15, 28. 28385155 10.1186/s12915-017-0365-7 PMC5383996

[pbi12903-bib-0043] Zhang, L. , Xu, Y. and Ma, R. (2008) Molecular cloning, identification, and chromosomal localization of two MADS box genes in peach (Prunus persica). J. Genet. Genomics, 35, 365–372. 18571125 10.1016/S1673-8527(08)60053-3

[pbi12903-bib-0044] Zhang, H. , Zhang, D. , Wang, M. , Sun, J. , Qi, Y. , Li, J. , Wei, X. *et al*. (2011) A core collection and mini core collection of Oryza sativa L. in China. Theor. Appl. Genet. 122, 49–61. 20717799 10.1007/s00122-010-1421-7

[pbi12903-bib-0045] Zhang, H. , Gao, S. , Lercher, M.J. , Hu, S. and Chen, W.H. (2012a) EvolView, an online tool for visualizing, annotating and managing phylogenetic trees. Nucleic Acids Res. 40, W569. 22695796 10.1093/nar/gks576 PMC3394307

[pbi12903-bib-0046] Zhang, X. , Wang, J. , Huang, J. , Lan, H. , Wang, C. , Yin, C. , Wu, Y. *et al*. (2012b) Rare allele of *OsPPKL1* associated with grain length causes extra‐large grain and a significant yield increase in rice. Proc. Natl. Acad. Sci. USA, 109, 21534–21539. 23236132 10.1073/pnas.1219776110 PMC3535600

[pbi12903-bib-0047] Zhang, F. , Yao, J. , Ke, J. , Zhang, L. , Lam, V.Q. , Xin, X.F. , Zhou, X.E. *et al*. (2015) Structural basis of JAZ repression of MYC transcription factors in jasmonate signalling. Nature, 525, 269–273. 26258305 10.1038/nature14661 PMC4567411

