# Supplementary material for: Alternative splicing of OsLG3b controls grain length and yield in japonica rice
Source: Plant Biotechnol J. 2018 Mar 24;16(9):1667–78. doi: 10.1111/pbi.12903 (PMC6097128; doi:10.1111/pbi.12903)
Supplement: Supplementary file 1 — Figure S1 (a) Grains from 10 typical tropical japonica varieties (CH1027, CH1029, CH1067, CH1091, CH1083, CH1085, CH1086, CH1058, IRAT109 and Haogelao), and 10 typical temperate japonica varieties (CH1001, CH1002, CH1004, CH1008, CH1009, CH1010, CH1020, CH1026, CH1071, and Nipponbare) (Table S5). Figure S2 Graphic genotype of BC4F3‐78‐11. Figure S3 Field trial of NIP and NIL(SLG) plants. Figure S4 Geographic origins of 266 indica and japonica rice accessions. Figure S5 Frequency distribution of grain length in the mini core collection (MCC population) (Yu et al., 2017). Figure S6 Amino acid sequence alignment of OsLG3b from Nipponbare (Nip), SLG and IRAT109. Figure S7 Grains from SLG, Nipponbare, and IRAT109. Scale bar, 5 mm. Figure S8 cDNA sequence alignment of OsLG3b from Nipponbare (Nip), SLG and IRAT109. Figure S9 The temporal‐spatial expression pattern of OsLG3b. Figure S10 Phenotypic analysis of CRISPR‐OsLG3b transgenic plants. Figure S11 Genotypes of OsLG3b in tropical japonica and indica or temperate japonica admixed with tropical japonica between landraces and improved varieties. Figure S12 Histograms showing distribution of grain length, grain width, length: width ratio and grain weight in temperate japonica (Tej) and tropical japonica (Trj) accessions. Figure S13 Phylogenetic tree of the representative wild rice accessions and sixteen indica or temperate japonica lines with the OsLG3b SLG allele. Figure S14 Comparison of grain lengths in large‐grain and small‐grain haplotypes for GS3 (a), GW8 (c), TGW6 (e) and OsLG3b (g) when Q structure (sub1, indica; sub2, japonica) exists. Figure S15 OsLG3b does not affect grain quality. Table S1 Means differences for the selected grain traits identified with t tests between temperate japonica and tropical japonica. Table S2 Identification of QTLs related to grain length, grain width, grain thickness and grain weight. Table S3 Analysis of polymorphisms at function variations’ sites between Nipponbare and SLG. Table S6 [file PBI-16-1667-s005.docx]

**Supplemental Information**

This PDF file includes:

Figs S1-S15

Tables S1-S3 and S6-S9

(Tables S4-S5 and S10-S12 are provided in the separate Excel files)

**Table of Contents**

**Figures**

- Fig. S1 Grains from 10 typical *tropical japonica* varieties.
- Fig. S2 Graphic genotype of BC_4_F_3_-78-11.
- Fig. S3 Field trial of NIP and NIL(SLG) plants.
- Fig. S4 Geographic origins of 266 rice accessions.
- Fig. S5 Frequency distribution of grain length in the mini core collection (MCC population).
- Fig. S6 Amino acid sequence alignment of *OsLG3b* from Nipponbare (Nip), SLG and IRAT109.
- Fig. S7 Grains from SLG, Nipponbare, and IRAT109. Scale bar, 5mm.
- Fig. S8 cDNA sequence alignment of *OsLG3b* from Nipponbare (Nip), SLG and IRAT109.
- Fig. S9 The temporal-spatial expression pattern of *OsLG3b*.
- Fig. S10 Phenotypic analysis of CRISPR*-OsLG3b* transgenic plants.
- Fig. S11 Genotypes of *OsLG3b* in *tropical japonica* and *indica* or *temperate japonica* admixed with *tropical japonica* between landrace and improved varieties.
- Fig. S12 Histogram showing distribution of grain length, grain width, length: width ratio and grain weight in *temperate japonica* accessions and *tropical* *japonica* accessions, respectively.
- Fig. S13 Phylogenetic tree of the representative wild rice accessions and sixteen *indica* or temperate *japonica* lines with the *OsLG3b^SLG^* allele.
- Fig. S14 Comparison of grain lengths in large-grain and small-grain haplotypes for *GS3* (a), *GW8* (c), *TGW6* (e) and *OsLG3b* (g) when Q structure (sub1, *indica*; sub2, *japonica*) exists.
- Fig. S15 *OsLG3b* does not affect grain quality.

**Tables**

- Table S1 Means differences for the selected grain traits identified with *t* tests between *temperate* *japonica* and *tropical* *japonica.*
- Table S2 Identification of QTLs related to grain length, grain width, grain thickness and grain weight.
- Table S3 Analysis of polymorphisms at function variations’ sites between Nipponbare and SLG.
- Table S4 Primers used for the genotyping of a near-isogenic line for the *qGL3-2* locus
- Table S5 Information of *Orayza sativa* L. varieties and wild rice on variety name, geographic source, stratification referred by STRUCTURE, the integrated stratification, grain lengths and allelic variations of grain-length-related genes
- Table S6 Summary of the taxa and source of 506 varieties.
- Table S7 Environments used to evaluate association and linkage populations.
- Table S8 The heritability of grain traits in MCC Panel.
- Table S9 *OsLG3b* polymorphisms associated with grain length in the MCC panel.
- Table S10 Thirty-nine SNPs in *OsLG3b* used in the introgression analysis.
- Table S11 Primers used for fine mapping and sequencing.
- Table S12 Primers used for DNA constructs and transcripts analysis.

**
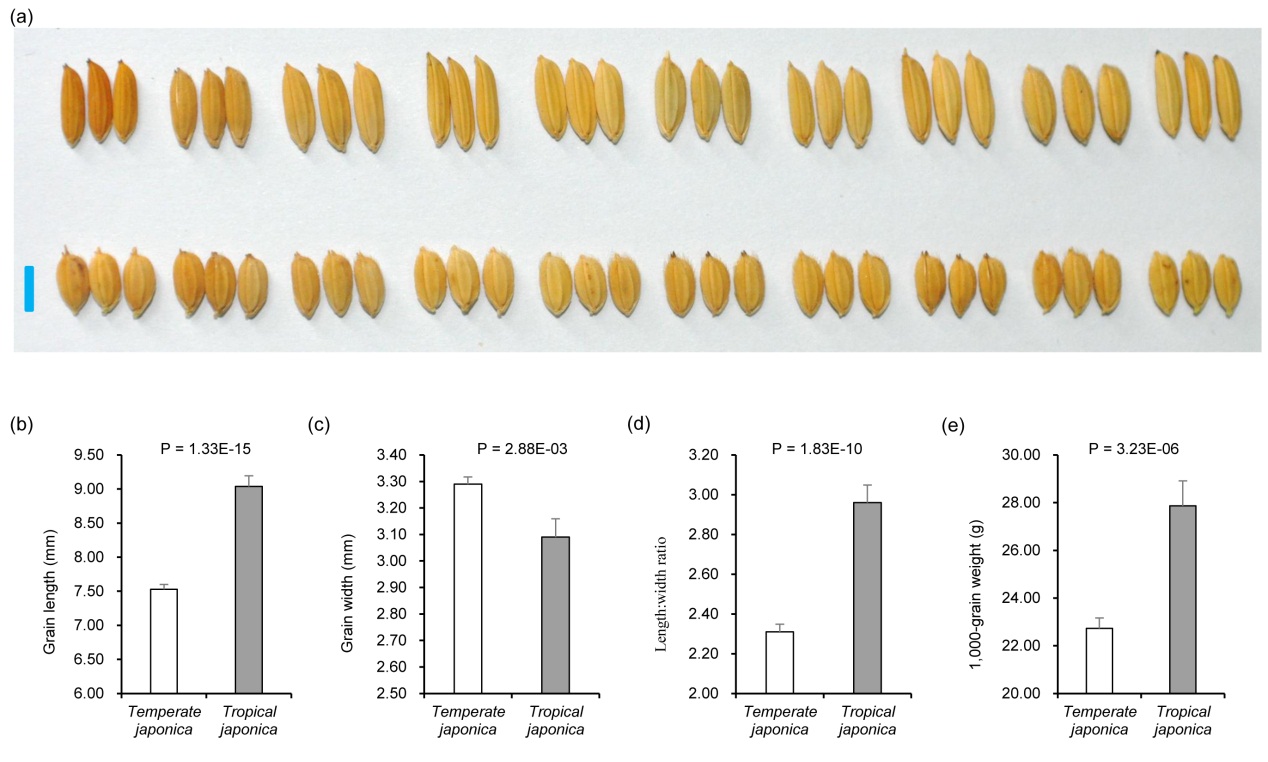
**

**Fig. S1**. (a) Grains from 10 typical *tropical japonica* varieties (CH1027, CH1029, CH1067, CH1091, CH1083, CH1085, CH1086, CH1058, IRAT109, and Haogelao), and 10 typical *temperate japonica* varieties (CH1001, CH1002, CH1004, CH1008, CH1009, CH1010, CH1020, CH1026, CH1071, and Nipponbare) (Table S5). Scale bar, 5mm. (b-e) Mean differences for the selected grain traits identified with *t* tests between *temperate* *japonica* (n=99) and *tropical* *japonica* (n=23)*.* All phenotypic data in b–e were measured from paddy-grown plants. Data represent means ± s.e.m. Student’s *t* tests were used to generate *P* values.

**
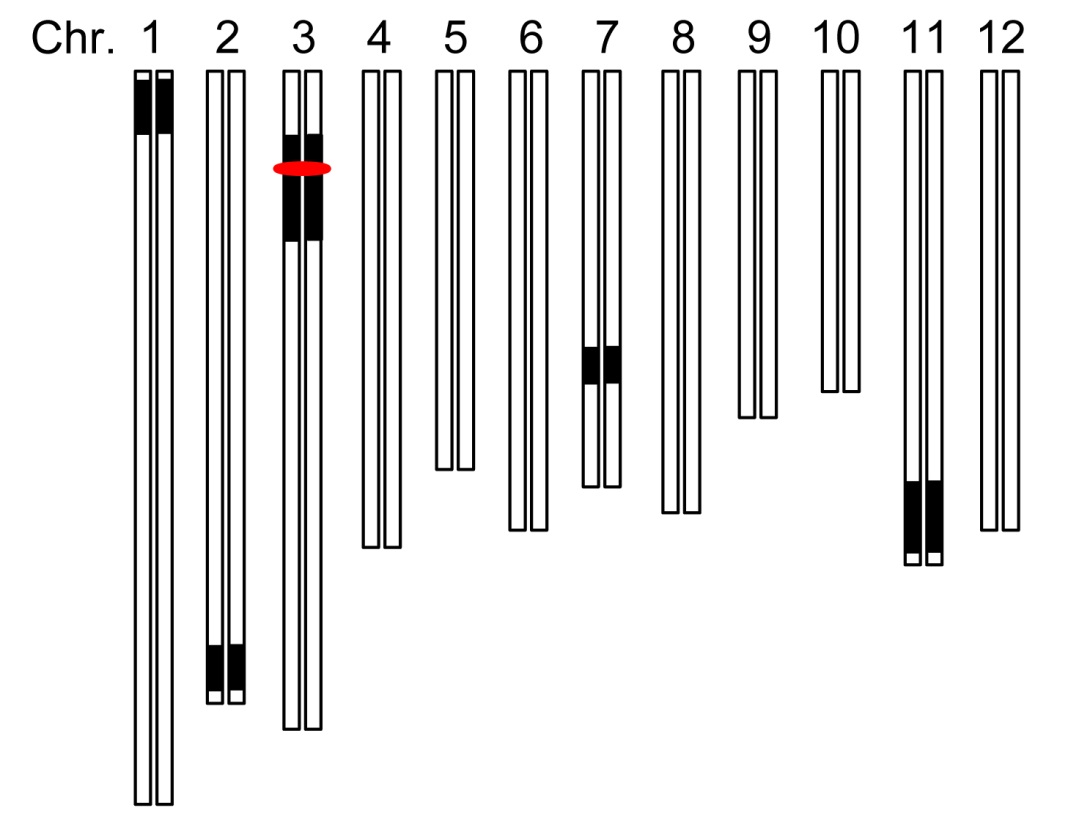
**

**Fig. S2.** Graphic genotype of BC_4_F_3_-78-11. The black regions indicate SLG introgressions. The white regions indicate NIP genetic background. The red ellipse indicates the *qGL3-2* locus. Based on the primary mapping results, plants homozygous for the *qGL3-2* locus were selected from more than 1,000 BC_4_F_3_ plants by SSR markers. When we surveyed them using ~100 SSR markers evenly distributed on all 12 rice chromosomes we identified plant BC_4_F_3_-78-11 that was homozygous for the target region and contained the least genetic background from SLG (about 9.5% of markers from SLG were homozygous) (Table S4).

**
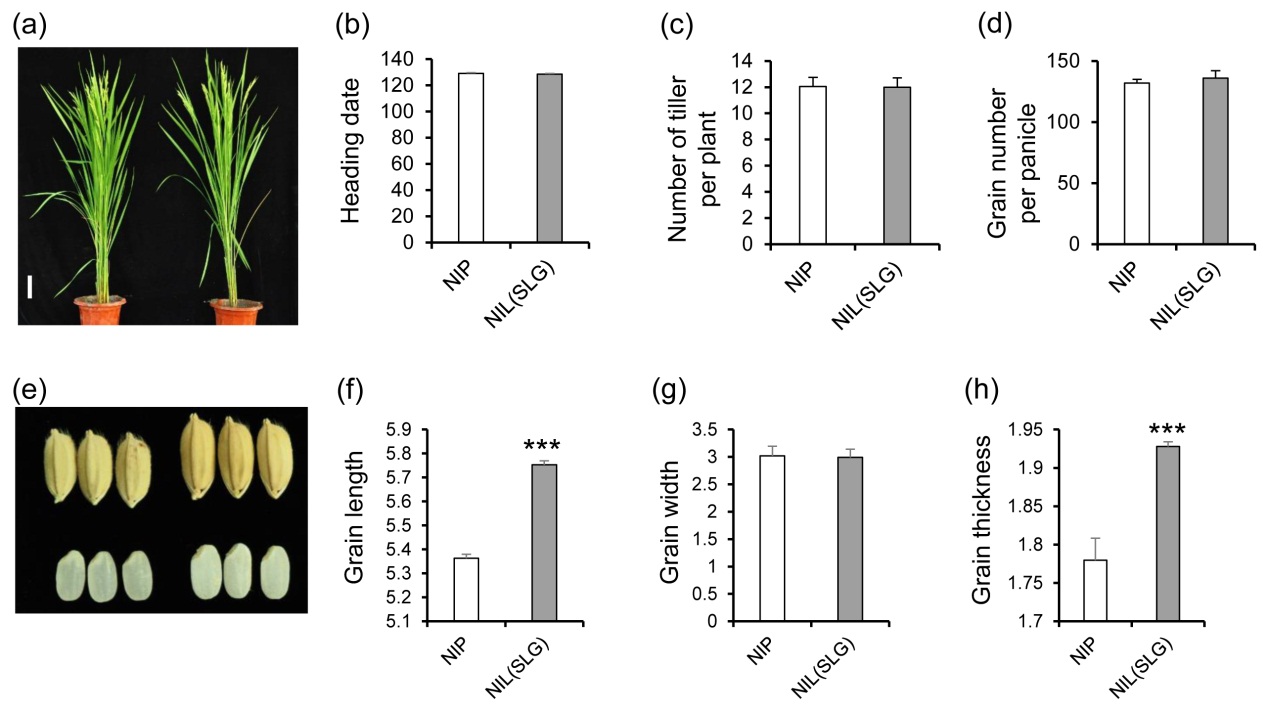
**

**Fig. S3.** Field trial of NIP and NIL(SLG) plants. (a) Morphologies of NIL plants. Scale bar, 10 cm. (b) Heading dates. (c) Number of tillers per plant. (d) Grain number per panicle. (e) Dehulled and hulled grains from the NILs. (f) Brown grain length. (g) Brown grain width. (h) Brown grain thickness. All phenotypic data in b–h were measured from paddy-grown plants. Data represent means ± s.e.m. (n = 30). Student’s *t* tests were used to generate *P* values.

**
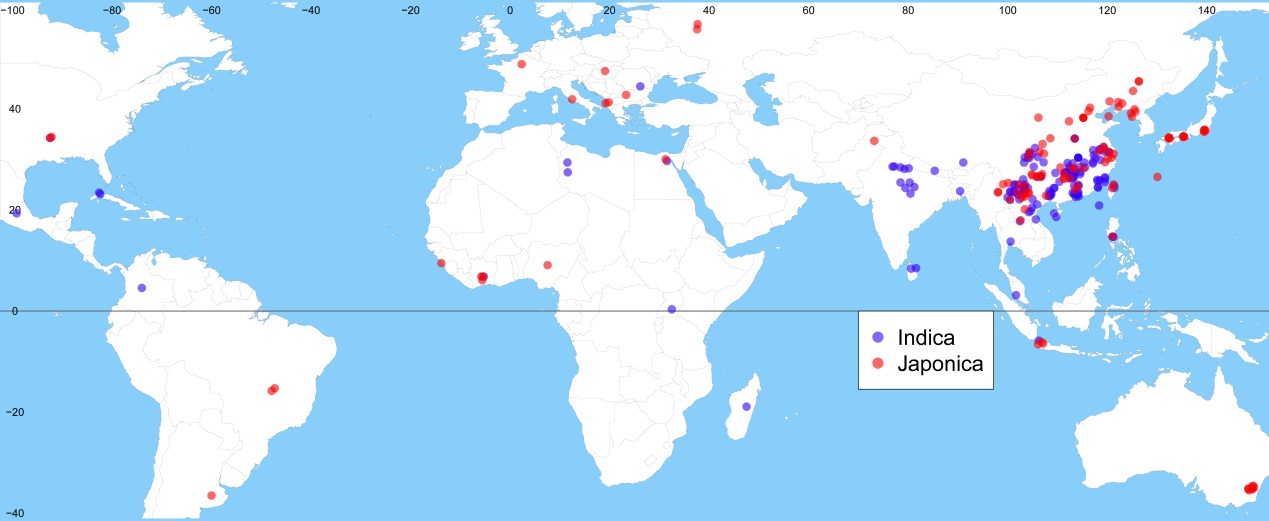
**

**Fig. S4.** Geographic origins of 266 *indica* and *japonica* rice accessions.

**
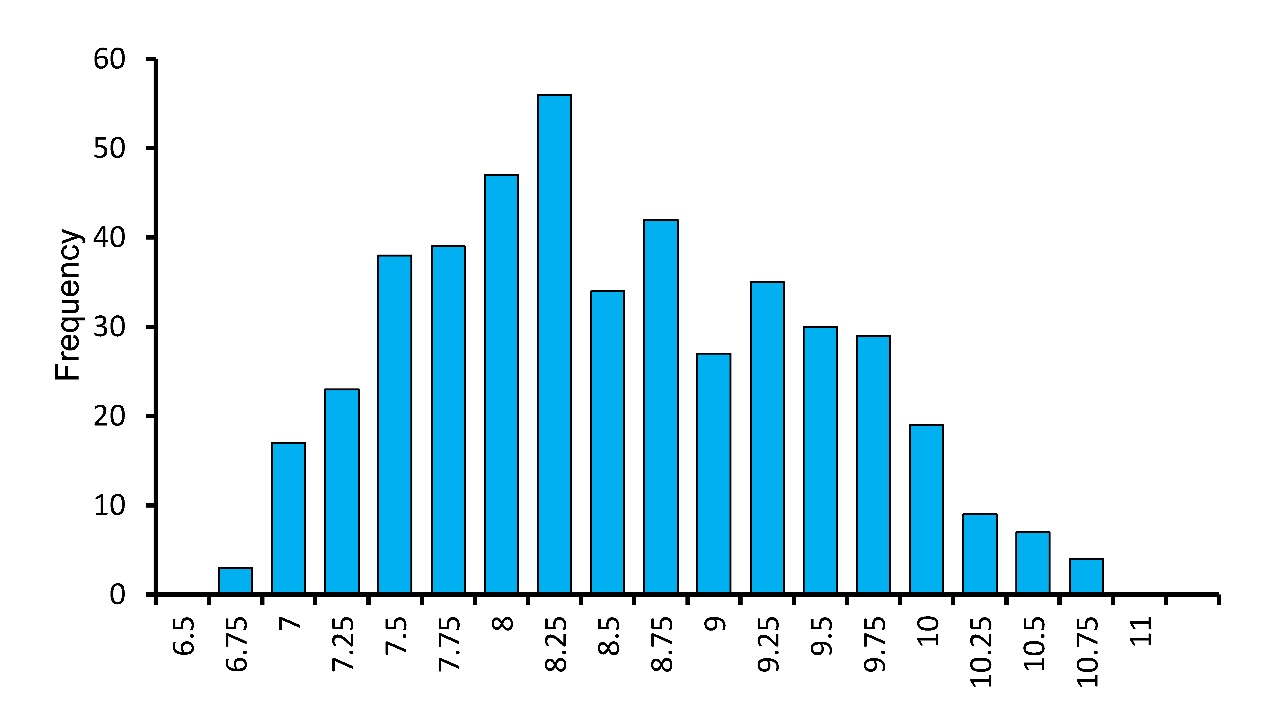
**

**Fig. S5.** Frequency distribution of grain length in the mini core collection (MCC population) (Yu et al., 2017).

**
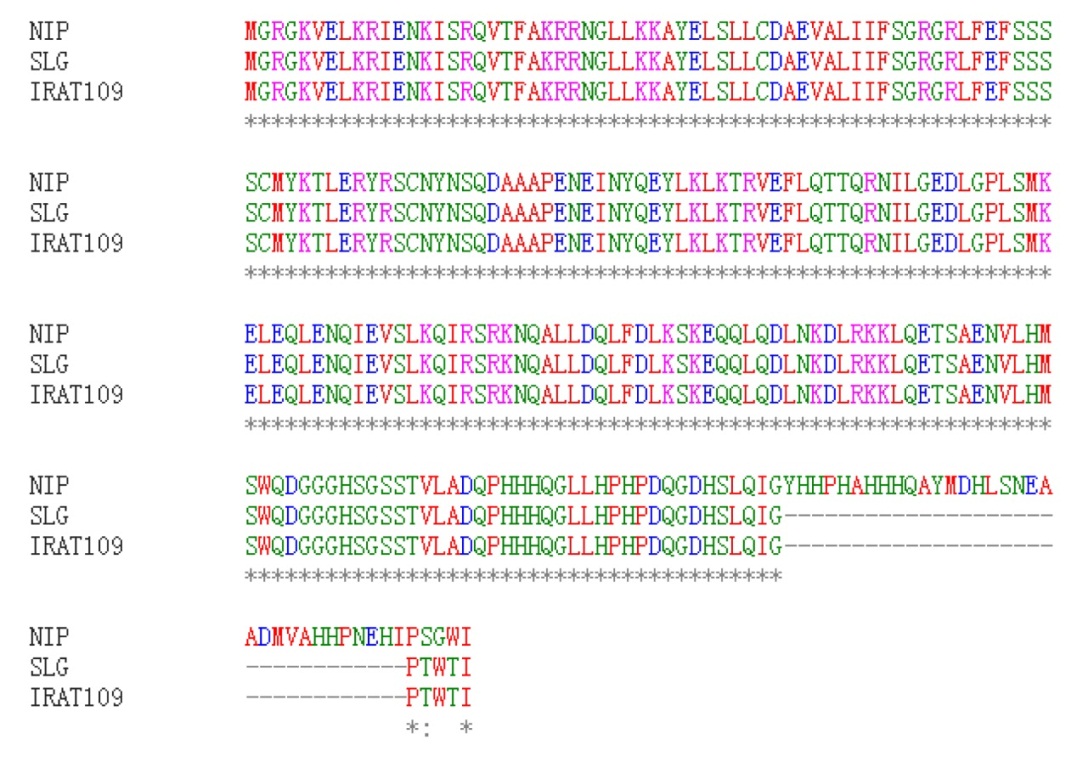
**

**Fig. S6.** Amino acid sequence alignment of *OsLG3b* from Nipponbare (Nip), SLG and IRAT109.


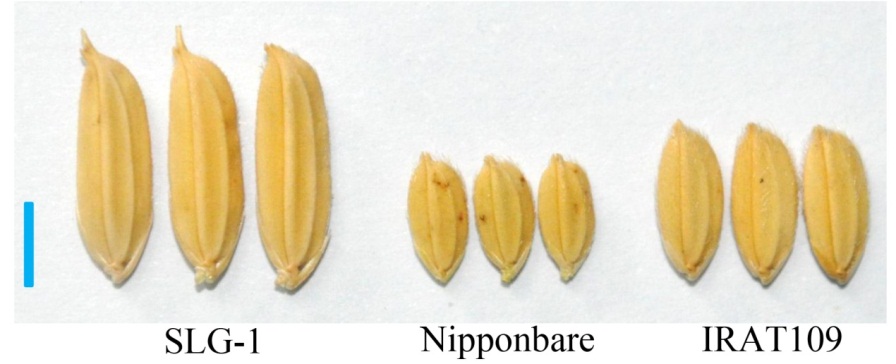


**Fig. S7.** Grains from SLG, Nipponbare, and IRAT109. Scale bar, 5mm.


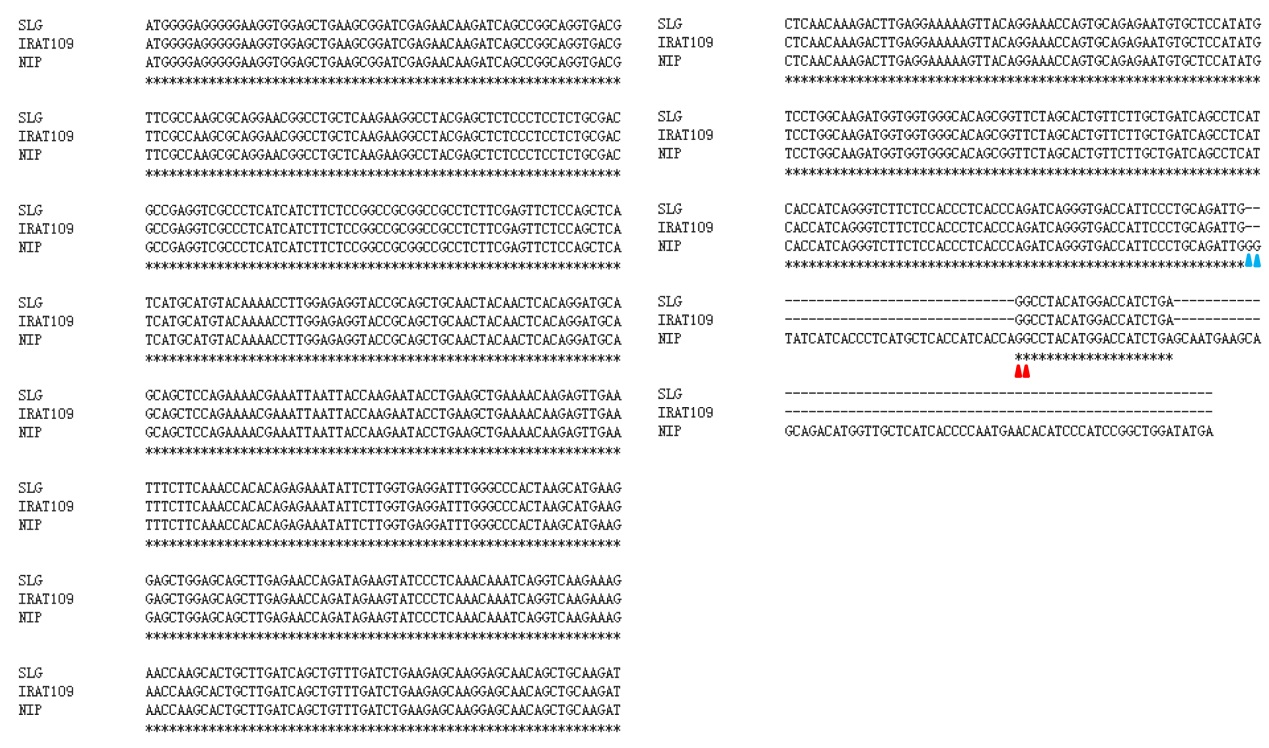


**Fig. S8.** cDNA sequence alignment of *OsLG3b* from Nipponbare (Nip), SLG and IRAT109. Blue triangles refer to the normal splice site; red triangle indicates the mutant splice site.


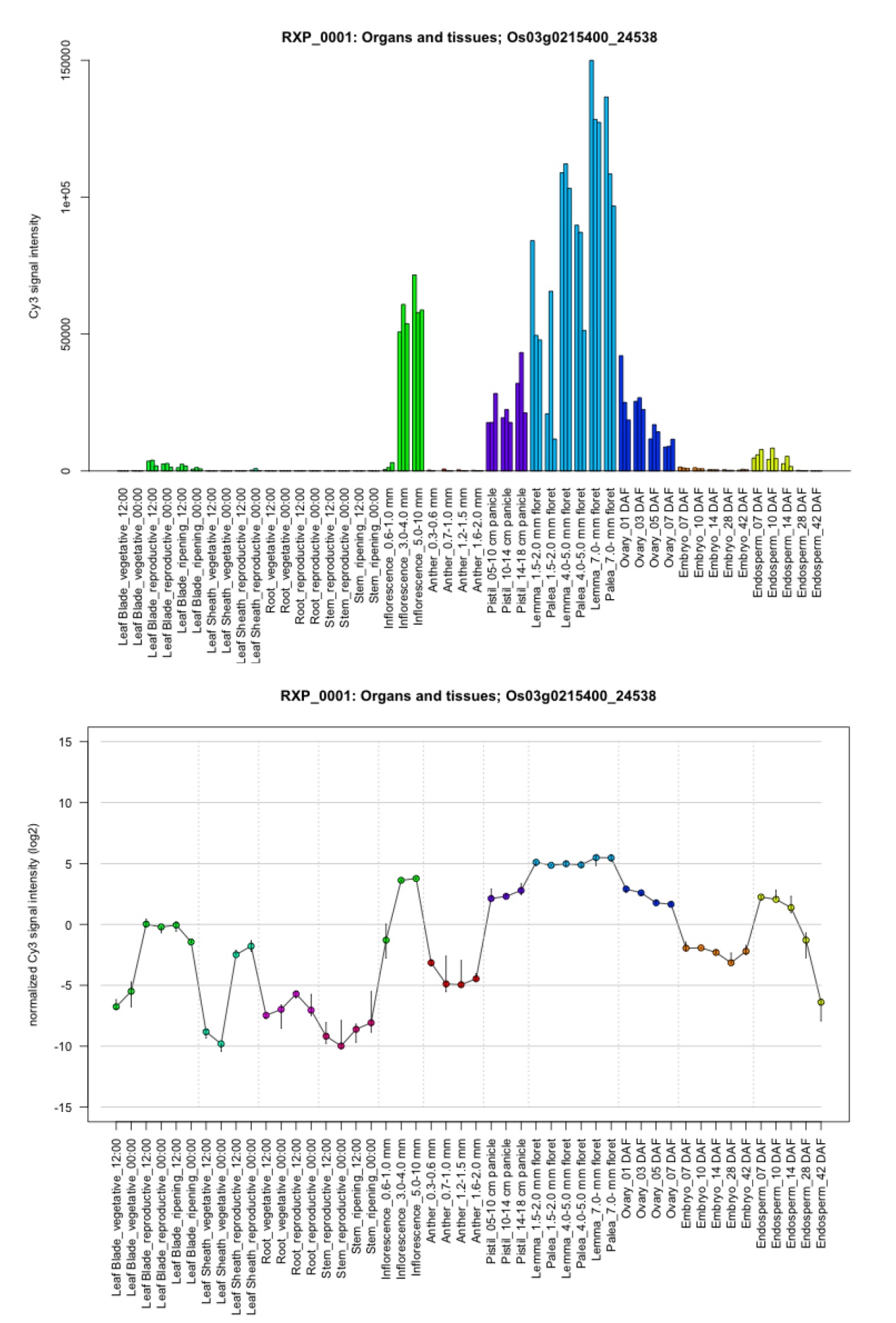


**b**

**a**


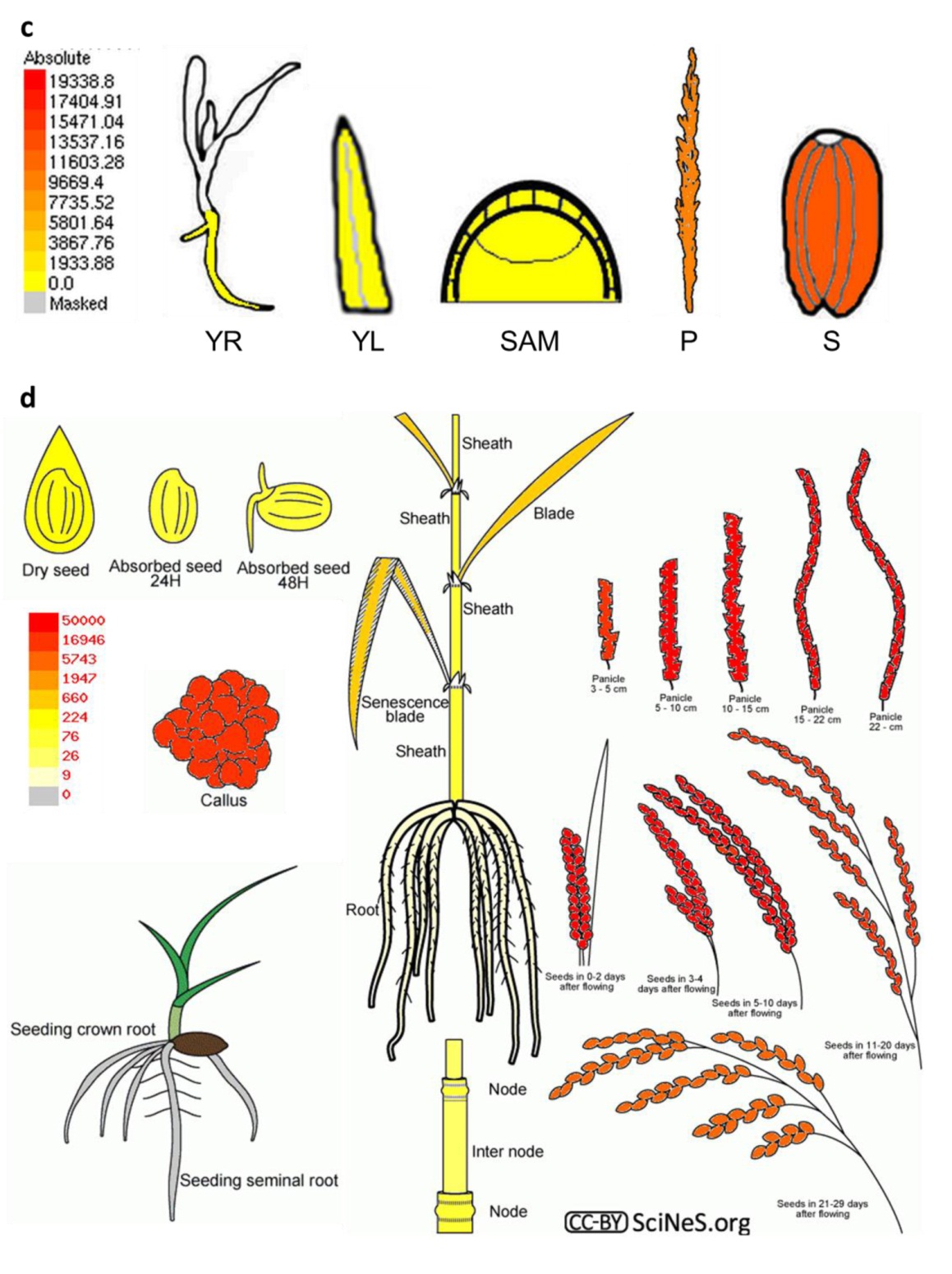


**Fig. S9.** The temporal-spatial expression pattern of *OsLG3b*. (**a, b**) Expression pattern of OsLG3b at different developmental stages based on microarray data displayed in the Rice Expression Profile Database (RiceXPro) (http://ricexpro.dna.affrc.go.jp/GGEP/). (**c**) Relative expression of OsLG3b in seedling roots (YR), young leaves (YL), shoot apical meristems (SAM), inflorescence (P) and seeds (S) based on microarray data displayed in the eFP browser (http://www.bar.utoronto.ca/efp/cgi-bin/efpWeb.cgi). Color scale shows microarray signal level. (**d**) Relative OsLG3b expression in different rice tissues as determined by microarray analysis using the eFP browser (HANDB-OS). Color scale indicates microarray signal levels.


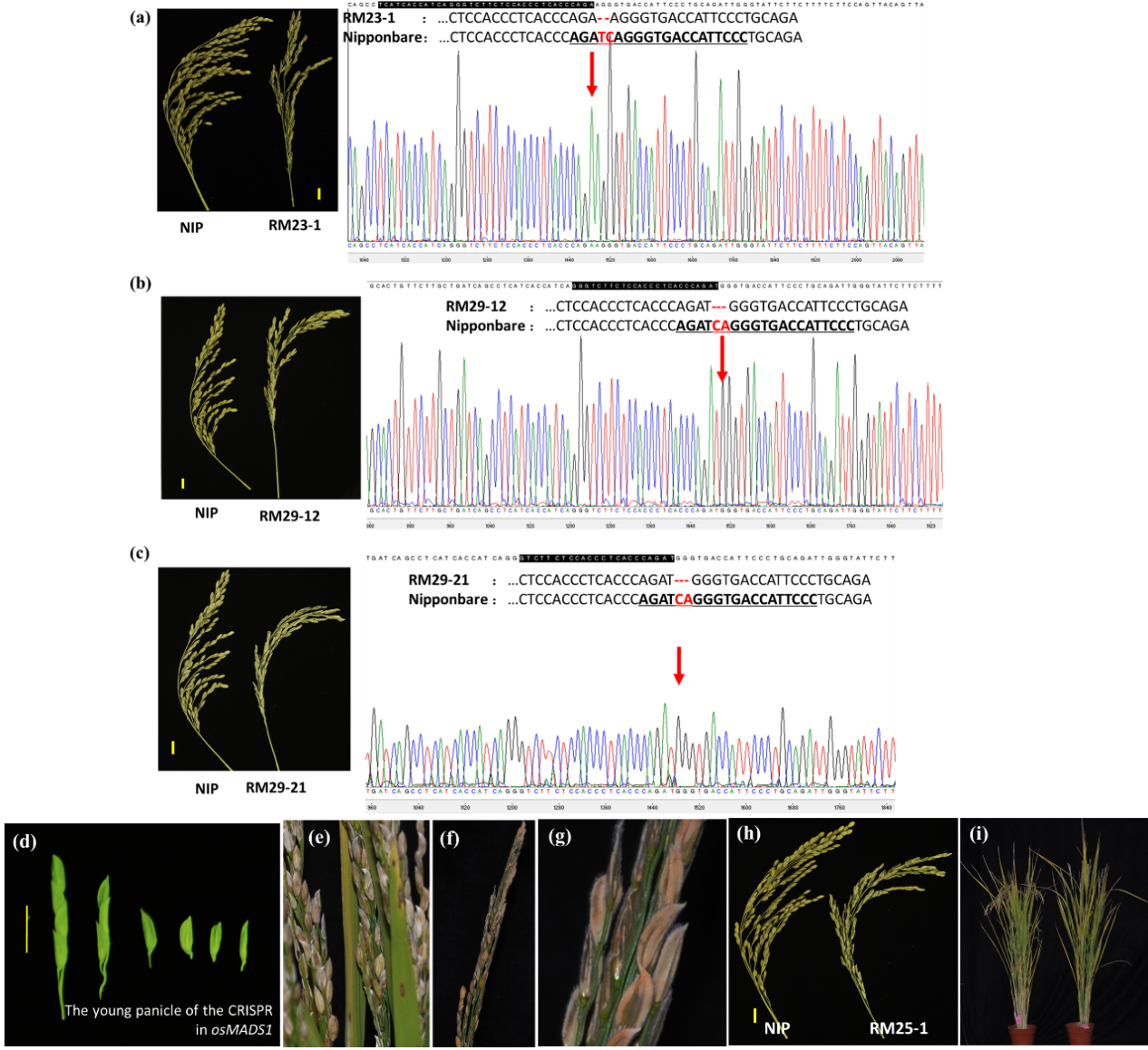


**Fig. S10.** Phenotypic analysis of CRISPR*-OsLG3b* transgenic plants. (**a-c**) The panicles from transgenic plants RM23-1, RM29-12 and RM29-21 (all homozygotous in the T1 generation, left) and their mutations in sequence (right), respectively. (**d**) Various morphologies of young spikelets before flowering. (**e-h**) Mature glumes and panicles of CRISPR*-OsLG3b* transgenic plants. (**i**) Mature plant appearance. Scale bars, 1.0 cm (yellow line); All plants were grown with 15×20 cm spacing in paddies under normal cultivation conditions.

**
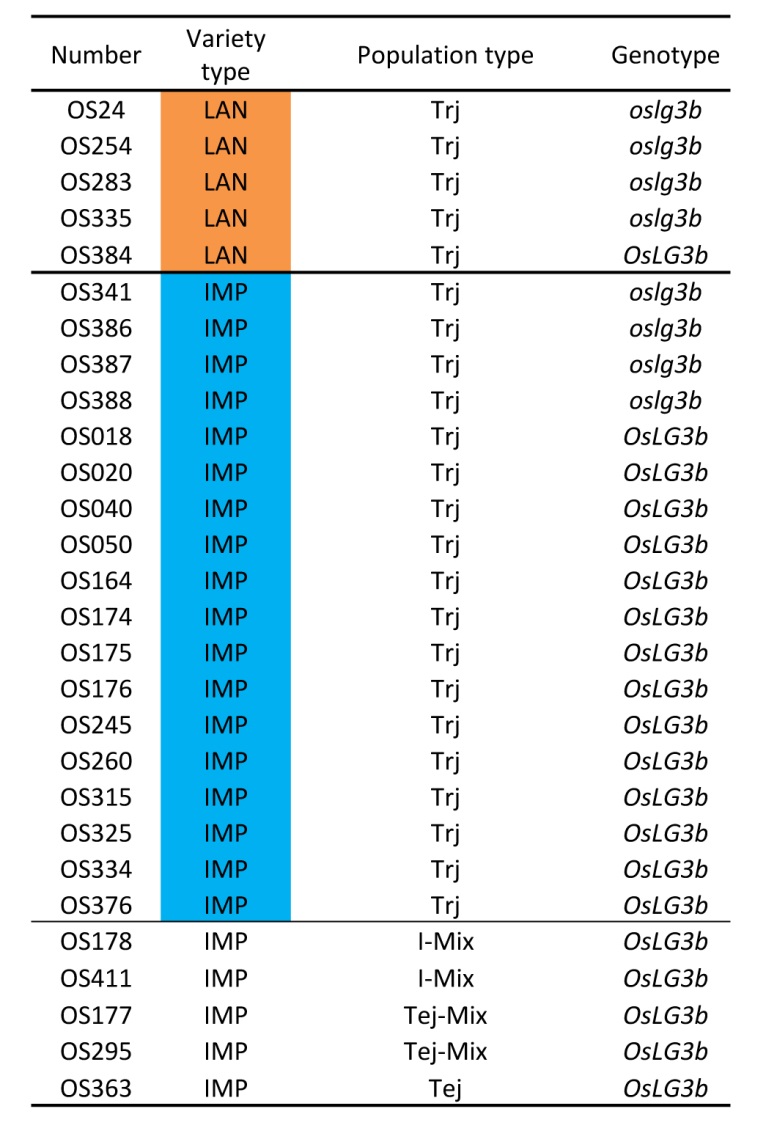
**

**Fig. S11.** Genotypes of *OsLG3b* in tropical *japonica* and *indica* or temperate *japonica* admixed with tropical *japonica* between landraces and improved varieties. LAN, landrace variety; IMP, improved variety; TRJ, tropical *japonica*; I-MIX, *indica* variety admixed with tropical *japonica* segments; TEJ-MIX, temperate *japonica* variety admixed with tropical *japonica* segments.


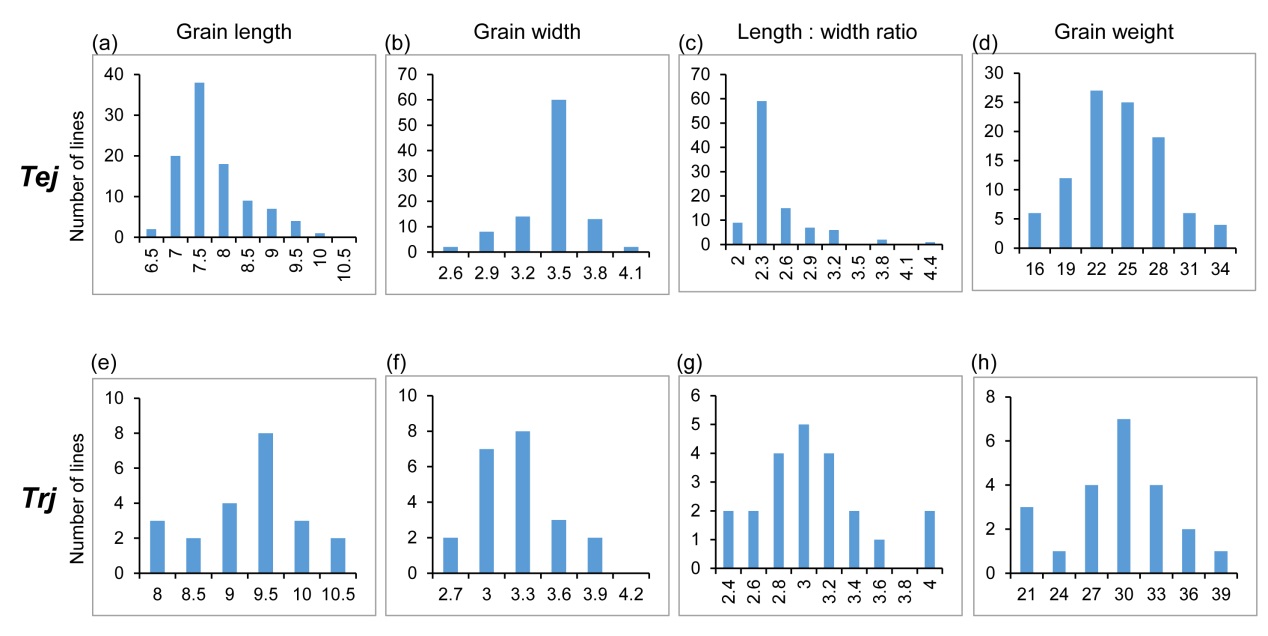


**Fig. S12.** Histograms showing distribution of grain length, grain width, length: width ratio and grain weight in temperate *japonica* (*Tej*) and tropical *japonica* (*Trj*) accessions. (a, e) Grain length; (b, f) Grain width. (c, g) Length: width ratio. (d, h) Grain weight.


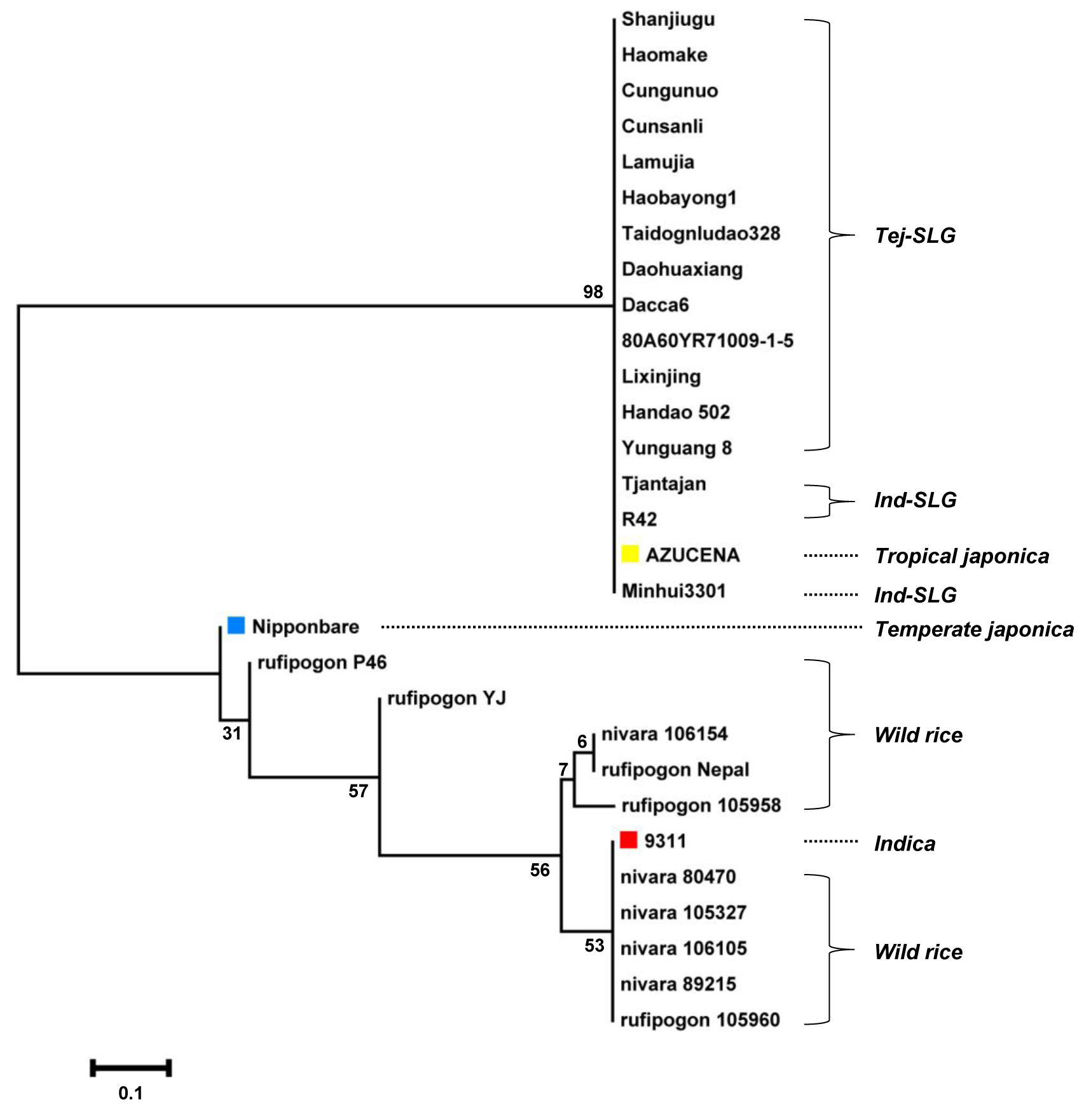


- **Fig. S13.** Phylogenetic tree of the representative wild rice accessions and sixteen *indica* or temperate *japonica* lines with the *OsLG3b^SLG^* allele. The phylogenetic tree was constructed based on 39 SNPs s in the proximal region of the *OsLG3b* gene (listed in Table S10) by MEGA 6.0. *Trj/Tej/I-SLG*, tropical/temperate *japonica* / *indica* accessions with the SLG-type *OsLG3b.*


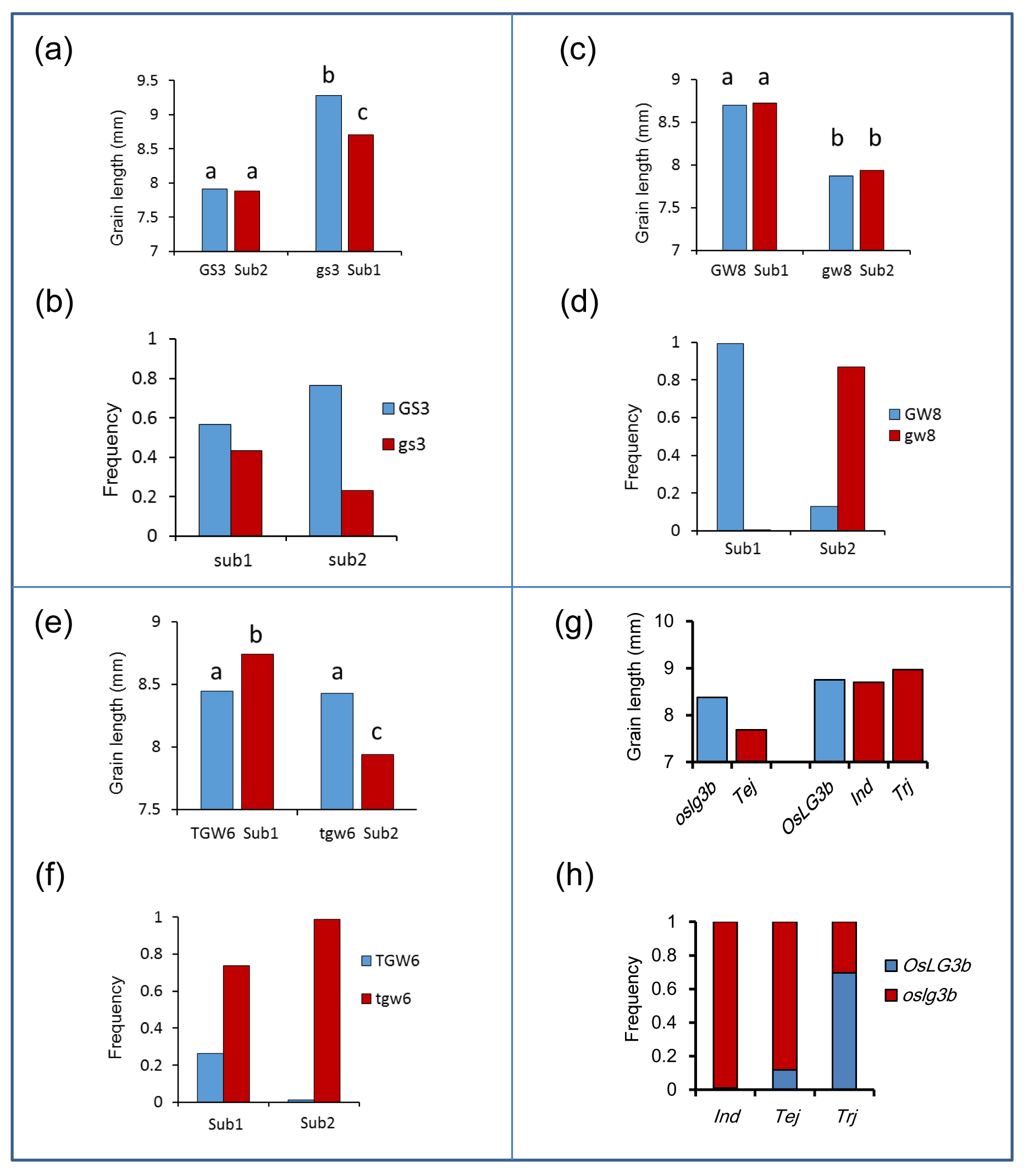


**Fig. S14.** Comparison of grain lengths in large-grain and small-grain haplotypes for *GS3* (a), *GW8* (c), *TGW6* (e) and *OsLG3b* (g) when Q structure (sub1, *indica*; sub2, *japonica*) exists. The bottom histogram of each box is frequency distribution between two genotypes in subpopulations about *GS3* (b), *GW8* (d), *TGW6* (f) and *OsLG3b* (h), respectively. The rate of each haplotype under two sub populations (sub1 and sub2) divided by Q for these genes. Ind, *indica*; Tej, *temperate japonica*; Trj, *tropical japonica*.


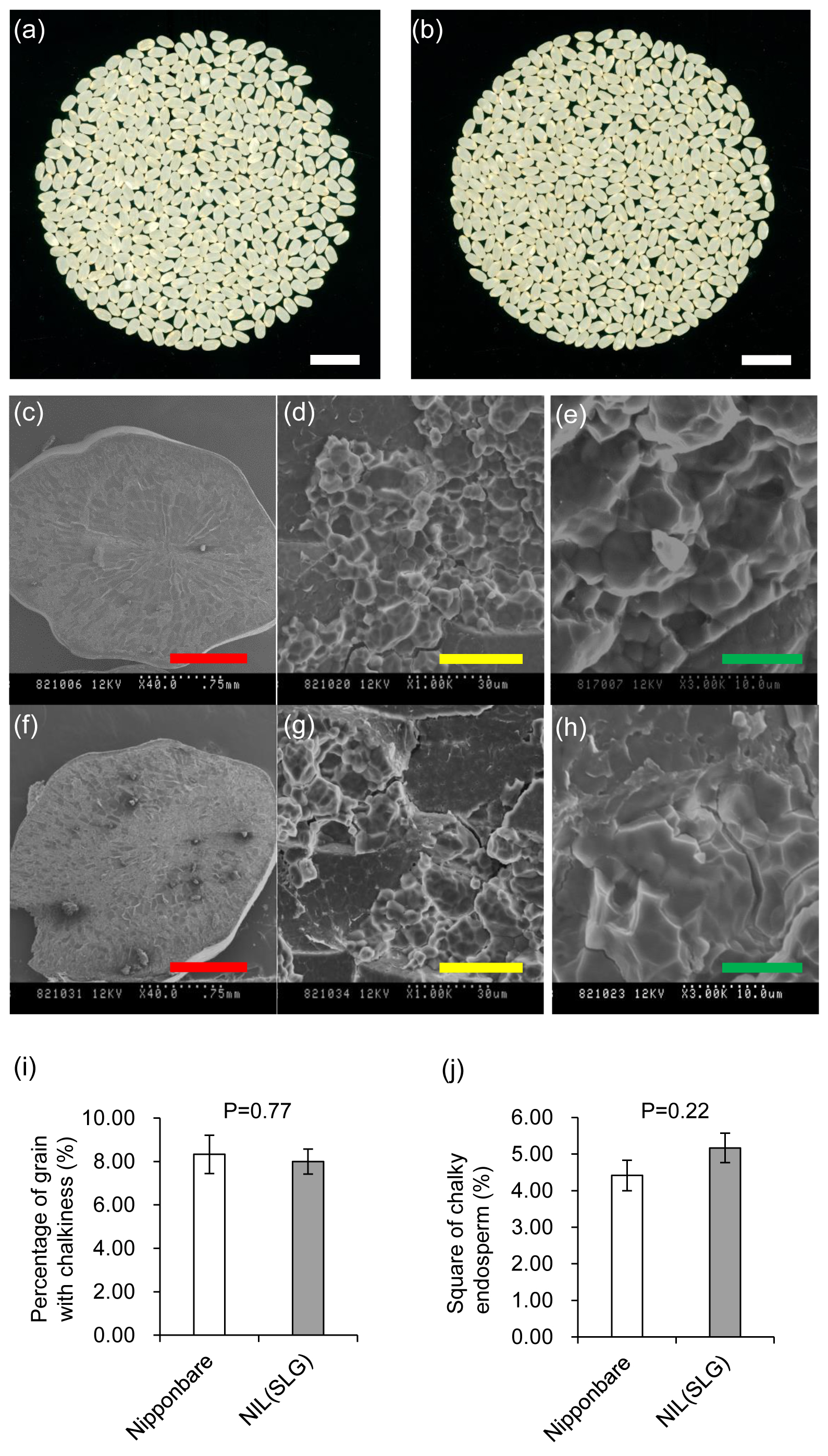


**Fig. S15** *OsLG3b* does not affect grain quality. (a, b) Comparison of brown grains NIP and NIL(SLG). Scale bar, 1 cm. (c-e) The scanning electron microscopy images are transverse sections of starch granule of Nipponbare, (f-h) The scanning electron microscopy images are transverse sections of starch granule of NIL(SLG). (i) Percentage of grain with chalkiness (%) (n=3), (j) Square of chalky endosperm (%) (n=3). Scale bars, 0.75 mm (red line), 30µm (yellow line) and 10µm (green line), respectively. Data are given as means ± SD. Student’s *t*-test was used to generate the *P* values.

**Tables**

(Tables S4 - S5 and S10-12 are provided in the separate Excel files)

**Table S1** Mean differences for the selected grain traits identified with *t* tests between *temperate* *japonica* and *tropical* *japonica.* The distributions of grain length, grain width, length:width ratio and grain weight in *temperate japonica* accessions and *tropical japonica* accessions are shown in Figure S12.

| Sub-population | Grain length (mm) | Grain width (mm) | Length: width ratio | 1,000-grain weight (g) |
| --- | --- | --- | --- | --- |
| *Temperate japonica* | 7.53 ± 0.07 | 3.29 ± 0.03 | 2.31 ± 0.04 | 22.73 ± 0.44 |
| *Tropical japonica* | 9.04 ± 0.15 | 3.09 ± 0.07 | 2.96 ± 0.09 | 27.87 ± 1.05 |
| *P* value | 1.33E-15 | 2.88E-03 | 1.83E-10 | 3.23E-06 |

**Table S2** Identification of QTLs related to grain length, grain width, grain thickness and grain weight.

| Trait | Chr. | QTL | Interval | LOD/*P* | PVE (%) | Loci reported |
| --- | --- | --- | --- | --- | --- | --- |
| Grain length | 2 | *qGL2-1* | RM3188-RM6378 | 5.41 | 12.34 |  |
|  | 2 | *qGL2-2* | RM6617-RM6366 | 2.70 | 6.00 |  |
|  | 3 | *qGL3-1* | GS09-RM6283 | 26.68 | 53.38 | *GS3* |
|  | 3 | *qGL3-2* | RM1278-RM3864 | 8.00 | 18.00 |  |
|  | 8 | *qGL8-1* | RM3452-RM5353 | 4.61 | 14.71 |  |
| Grain width | 2 | *qGW2-1* | RM3188-RM6378 | 12.27 | 25.84 | *GW2* |
|  | 2 | *qGW2-2* | RM5651-RM1367 | 2.61 | 6.81 |  |
|  | 5 | *qGW5-1* | RM7653-RM3809 | 2.76 | 8.32 |  |
| Grain thickness | 3 | *qGT3-2* | RM1278-RM3864 | 7.30 | 17.20 |  |
| 1,000-grain weight | 2 | *qTGW2-1* | RM3188-RM6378 | 5.41 | 12.34 |  |
|  | 3 | *qTGW3-2* | RM5477-RM14682 | 5.79 | 13.42 |  |
|  | 3 | *qTGW3-1* | GS09-RM6283 | 13.63 | 32.29 |  |
|  | 6 | *qTGW6-1* | RM3370-RM1161 | 2.64 | 6.71 | *TGW6* |

Note: Chr., chromosome; LOD, log-likelihood value; PVE, phenotypic variation explained by QTL; qGL, QTL for grain length; qGT, QTL for grain thickness; qGW, QTL for grain width; qTGW, QTL for 1,000-grain weight. The relevant primer sequences are listed in Table S4.

**Table S3** Polymorphisms between Nipponbare and SLG at functional sites.

| Gene | Site of FNP | Variation type | | Change in protein |
| --- | --- | --- | --- | --- |
|  |  | Nipponbare | SLG |  |
| *GS3* | C165A | C | A | Premaure stop |
| *GW2* | A316 | AAAA | AAA | Premaure stop |
| *TGW6* | FNP313 | G | - | Premaure stop |

**Table S6** Summary of taxa and source of 506 *Oryza sativa* varieties (Yu et al., 2017).

| Source | Type | *Indica* | *Japonica* | *Adm* | *Aus* | *Aromatic* | Total |
| --- | --- | --- | --- | --- | --- | --- | --- |
| China | Landrace | 64 | 45 | 6 | 1 | 0 | 116 |
|  | Improved | 138 | 60 | 6 | 1 | 0 | 205 |
| Foreign | Landrace | 27 | 28 | 2 | 1 | 6 | 64 |
|  | Improved | 77 | 32 | 6 | 2 | 4 | 121 |
| Total |  | 306 | 165 | 20 | 5 | 10 | 506 |

Note: Adm, admixed subpopulation; Landrace, landrace variety; Improved, improved variety.

**Table S7.** Environments used to evaluate association and linkage populations (Yu et al., 2017).

| Population |  | Environment | Longitude and latitude | Mean day lengths (h) |
| --- | --- | --- | --- | --- |
| Association panel | MCC | Sanya, Hainan, 2010 | E109.3 , N18.1 | 10.3 |
|  | MCC | Sanya, Hainan, 2012 | E109.3 , N18.1 | 10.3 |
|  | MCC | Changsha, Hunan, 2013 | E112.6 , N28.1 | 13.3 |
|  | MCC | Sanya, Hainan, 2013 | E109.3 , N18.1 | 10.3 |
|  | MCC+MCC2 | Sanya, Hainan, 2014 | E109.3 , N18.1 | 10.3 |
| Linkage populations |  | Beijing, 2012 | E116.2 , N39.5 | 14.7 |
|  |  | Sanya, Hainan, 2012 | E109.3 , N18.1 | 10.3 |
|  |  | Beijing, 2013 | E116.2 , N39.5 | 14.7 |

**Table S8.** Heritabilities of grain traits in the MCC panel (Yu et al., 2017).

| Trait | Broad-sense heritability (%) |
| --- | --- |
| Grain length | 88 |
| Grain width | 90 |
| Grain thickness | 82 |
| Grain weight | 97 |

**Table S9.** *OsLG3b* polymorphisms associated with grain length in the MCC panel.

| Polymorphic site | R^2^(%) ^a^ | P ^b^ | Ho-index ^c^ | Location |
| --- | --- | --- | --- | --- |
| snp1 | 10.3 | 1.59E-14 | 1 | Exon 8 |
| snp2 | 10.6 | 9.19E-15 | 1 | Intron 7 |
| snp3 | 10.3 | 1.12E-13 | 1 | Intron 7 |
| snp4 | 10.6 | 1.20E-14 | 1 | Intron 7 |
| snp5 | 10.2 | 5.85E-11 | 1 | Intron 7 |
| snp6 | 9.94 | 1.15E-10 | 1 | Intron 7 |

^a^ R^2^ values from ANOVA of grain length showing % phenotypic variation explained.

^b^ p-value from candidate region association analysis for *qGL3-2*.

^c^ Ho (observed heterozygousity per locus) index (Yu et al., 2017) as the ratio between the number of heterozygotes corresponding to each SNP locus and the total number of crosses containing detected targeted QTL.
